# Localized Hotspots Drive Continental Geography of Abnormal Amphibians on U.S. Wildlife Refuges

**DOI:** 10.1371/journal.pone.0077467

**Published:** 2013-11-18

**Authors:** Mari K. Reeves, Kimberly A. Medley, Alfred E. Pinkney, Marcel Holyoak, Pieter T. J. Johnson, Michael J. Lannoo

**Affiliations:** 1 Anchorage Field Office, United States Fish and Wildlife Service, Anchorage, Alaska, United States of America; 2 Ecology and Evolutionary Biology, University of Colorado, Boulder, Colorado, United States of America; 3 Chesapeake Bay Field Office, United States Fish and Wildlife Service, Annapolis, Maryland, United States of America; 4 Environmental Science and Policy, University of California, Davis, Davis, California, United States of America; 5 Anatomy and Cell Biology, Indiana University School of Medicine, Terre Haute, Indiana, United States of America; Gettysburg College, United States of America

## Abstract

Amphibians with missing, misshapen, and extra limbs have garnered public and scientific attention for two decades, yet the extent of the phenomenon remains poorly understood. Despite progress in identifying the causes of abnormalities in some regions, a lack of knowledge about their broader spatial distribution and temporal dynamics has hindered efforts to understand their implications for amphibian population declines and environmental quality. To address this data gap, we conducted a nationwide, 10-year assessment of 62,947 amphibians on U.S. National Wildlife Refuges. Analysis of a core dataset of 48,081 individuals revealed that consistent with expected background frequencies, an average of 2% were abnormal, but abnormalities exhibited marked spatial variation with a maximum prevalence of 40%. Variance partitioning analysis demonstrated that factors associated with space (rather than species or year sampled) captured 97% of the variation in abnormalities, and the amount of partitioned variance decreased with increasing spatial scale (from site to refuge to region). Consistent with this, abnormalities occurred in local to regional hotspots, clustering at scales of tens to hundreds of kilometers. We detected such hotspot clusters of high-abnormality sites in the Mississippi River Valley, California, and Alaska. Abnormality frequency was more variable within than outside of hotspot clusters. This is consistent with dynamic phenomena such as disturbance or natural enemies (pathogens or predators), whereas similarity of abnormality frequencies at scales of tens to hundreds of kilometers suggests involvement of factors that are spatially consistent at a regional scale. Our characterization of the spatial and temporal variation inherent in continent-wide amphibian abnormalities demonstrates the disproportionate contribution of local factors in predicting hotspots, and the episodic nature of their occurrence.

## Introduction

**Figure 1:**
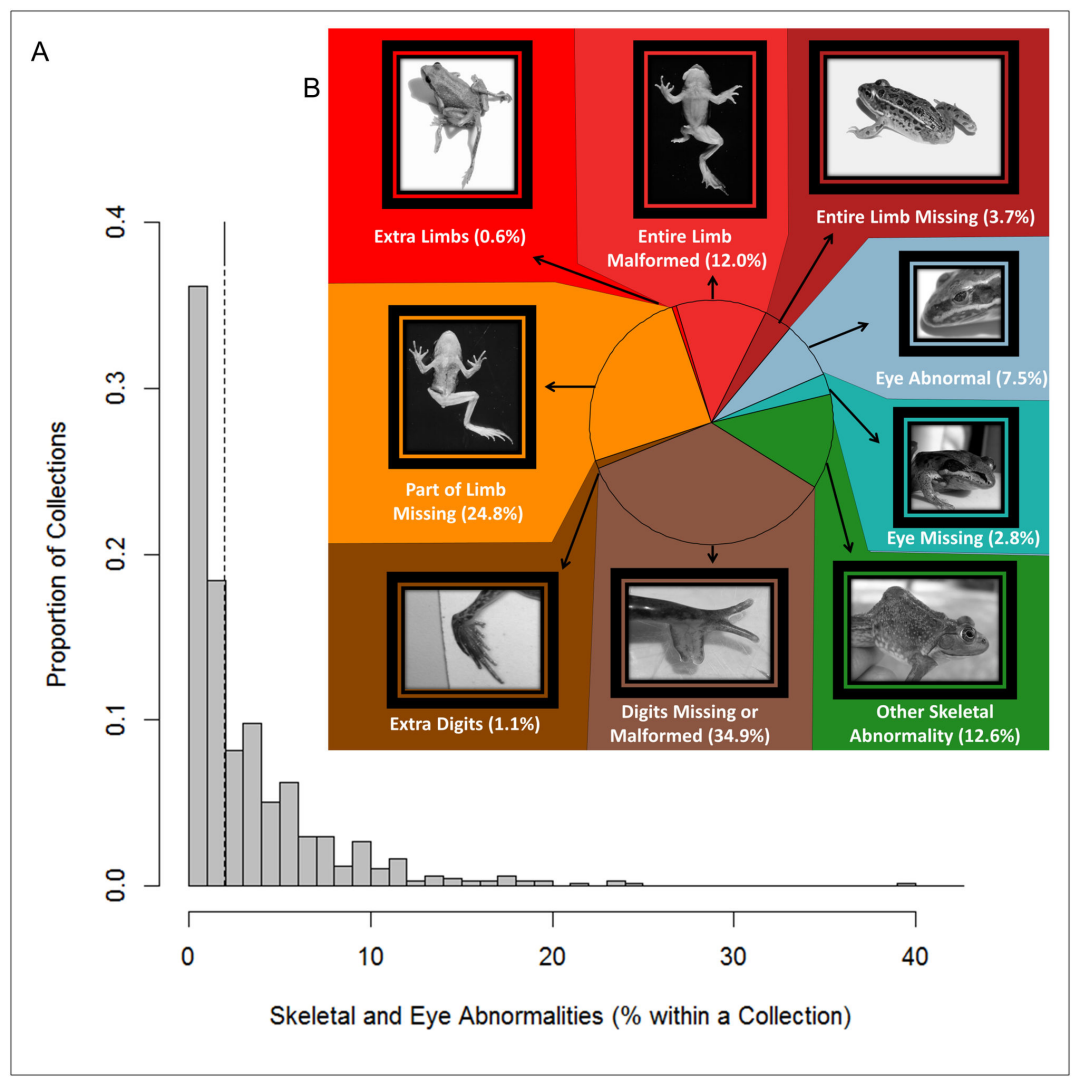
Description of field survey results. (A) Percentages of abnormal frogs in individual collection events (sampling of at least 30 individuals of one species at one site from the core dataset of 48,081 amphibians (Text S1)). Dashed line is estimate of national mean frequency (2%, CI_95%_ = 1.8–2.2%). (B) Percentages in photo figure are proportions of each abnormality out of total abnormalities (Table S3). Red shading indicates abnormality affected entire limb, orange indicates part of limb, brown indicates only the digits, green indicates abnormality affected parts of the skeleton other than limbs, and blue indicates eye abnormalities. Photo credits: extra limbs (D. Herasimtschuk), entire limb malformed and part of limb missing (D. Green), remainder (USFWS).

In North America, widespread observations of abnormal amphibians, including frogs with missing, misshapen, and extra limbs (Figure 1), garnered scientific and media attention beginning in the 1990s [1,2]. In the last two decades, regional-scale studies have identified populations with high abnormality frequencies—hotspots, operationally defined as sites exceeding 5% abnormal amphibians [2-4]. Several authors have presented evidence that abnormalities are increasing through time [5-7], that some species are especially susceptible [8], and that abnormal frogs may indicate poor environmental quality ([9], but also see 10). Nevertheless, a lack of knowledge of the broad spatial distribution and temporal dynamics of amphibian abnormalities has hindered efforts to understand the extent of the problem and the appropriate scale at which to evaluate its causes and implications. Amphibians are the most imperiled class of vertebrates on earth [11,12], and the abnormalities themselves or the factors that cause them may contribute to amphibian population declines [13-15]. 

Reported causes of amphibian abnormalities include parasite infection, injury by predators, chemical contaminants, ultraviolet-B radiation (UVB), and interactions among these (Table S1). Although the evidence for some of these single-factor causes is compelling [14,16-22], there has been substantial debate about their generality. For example, although some aquatic predators (e.g., larval dragonflies, small fishes, and leeches) have been linked to amphibian abnormalities [17,18], such abnormalities can be rare or absent even at sites with high predator abundances [17,18,23-25]. Similarly, infection by the trematode parasite *Ribeiroia ondatrae* can cause limb malformations in a wide range of amphibian species [26], and has been linked to malformation hotspots in the Western, Midwestern, and Eastern United States [2,14,27-29]. However, at sites without infection or those dominated by missing-limb abnormalities reported in a number of areas, infection by *Ribeiroia* cannot explain observed patterns [2,30-32]. Others have suggested a link between abnormalities and environmental contaminants, either directly as teratogens or indirectly through their effects on predators or parasites [20,27,32-36]. UVB as a single-factor hypothesis [22] has been largely disputed by research suggesting it is implausible as a solitary driver for abnormalities in the field [37,38], yet a recent study implicated UVB as a cofactor, correlating with increased abnormality frequencies when contaminants were also present [35]. It remains unclear whether single causal factors vary among samples and studies, or whether multiple causal factors commonly operate together in nature. Because multiple causal factors can operate at distinct spatial or temporal scales, a critical step forward is to characterize broad-scale pattern and variation in abnormality frequencies in amphibian populations. 

Broad-scale pattern analysis provides a useful, and sometimes critical, first step for understanding processes governing ecological and disease dynamics. Indeed, spatial epidemiology is widely used to characterize patterns of disease incidence and spread, and can lead to testable hypotheses about causation [39]. For instance, spatiotemporal analysis of disease incidence data has allowed researchers to propose better strategies for managing pathogens targeting high value crops, leading to more effective control [40]. Unfortunately, most field surveys for abnormal amphibians in North America prior to the one we report have occurred at the local to regional scale, and most have focused primarily on known hotspot locations (for example, in the Northeast [41], Minnesota [42], Alaska [31], and the Western United States [16]). Such targeted investigations are useful for determining the scope of the issue and its causes within a particular hotspot, but this approach does not address the broader spatial distribution of abnormalities, nor whether hotspots occur outside of these well-studied areas. The information gained in such a large-scale, systematic, spatial analysis allows us to identify areas where abnormalities are unusually elevated, and thus shed light on the scope of the problem in ways that investigations targeted solely at hotspots cannot.

Here, we combine a large-scale field survey with multiple analytical approaches to characterize spatial and temporal variation in amphibian abnormality frequencies, and describe the spatial and temporal scales at which they occur. Between 2000 and 2009, we assessed abnormalities in 62,947 individual amphibians from 497 wetland sites on 135 U.S. Fish and Wildlife Service (USFWS) National Wildlife Refuges to characterize the geographic distribution of skeletal and eye abnormalities. Our data allowed us to estimate an overall mean abnormality frequency for lands managed for wildlife conservation, and to evaluate the magnitude of variation contributed by space, time, and amphibian species. We further examined whether abnormalities clustered spatially, and if so, at what scales. Lastly, because year-to-year variation in abnormalities within sites was substantial, we tested whether temporal variation within spatially defined hotspot clusters differed relative to other areas. Our characterization of the spatial and temporal patterns in amphibian abnormalities on USFWS Refuge lands should help guide future investigations and improve land management in hotspot areas.

## Results

### Overview of patterns of abnormality occurrence

Amphibians with skeletal and eye abnormalities occurred infrequently on USFWS Refuge lands, based on our analysis of 48,081 amphibians representing 32 species of frogs and toads and 462 wetland sites (*core dataset, Methods*). One-third of the 675 collection events yielded no abnormal amphibians, and half of collections had fewer than 2% abnormal individuals (Figure 1, Table S2). Estimated mean abnormality frequency was 2% (95% confidence interval (CI_95%_) = 1.8–2.2%, n=675 collections and 48,081 individuals), yet abnormalities were highly aggregated in both space and time, with up to 40% abnormal amphibians at a site. Thus, while over 75% of collections fell below the generally accepted 5% hotspot classification criterion [2-4], 152 collection events yielded frequencies higher than this, with a long tail in the overall distribution (representing samples with high abnormality frequencies relative to the bulk of the distribution; Figure 1, Table S2). The most commonly observed abnormalities involved missing or shortened elements in the digits or limbs (Figure 1, Table S3). Notably, the severe and grotesque extra-limb abnormalities (polymelia) that garnered media attention in the 1990s were exceedingly rare on USFWS lands managed for wildlife conservation, comprising just 12 polymelic individuals (0.6% of all abnormalities, 0.025% all frogs in the *core dataset*; Figure 1, Table S3). 

### Spatial patterns in abnormality frequencies

**Figure 2:**
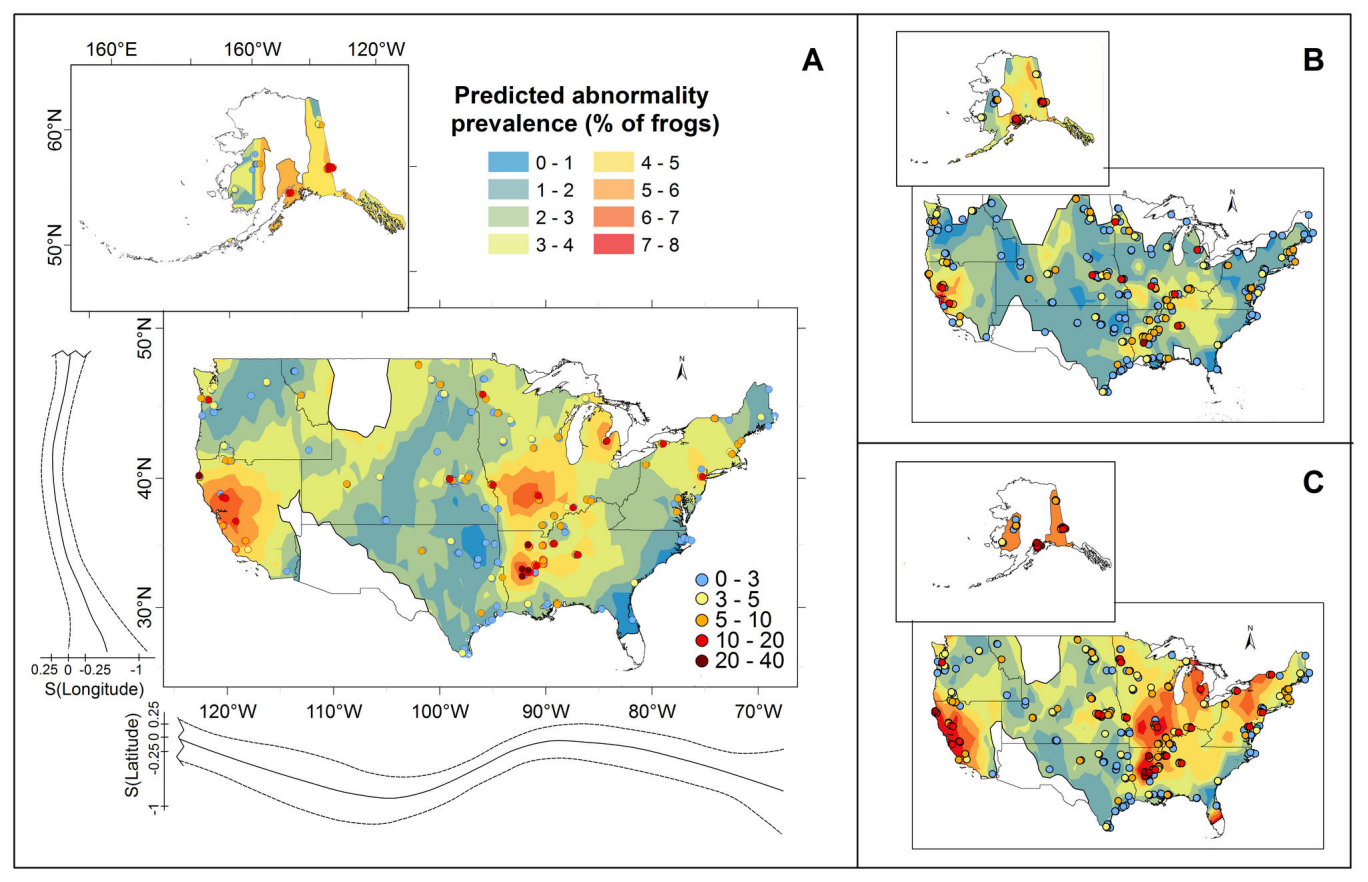
Geographic distribution of amphibian abnormalities with hotspot clusters. Warmer colors represent higher predicted abnormality prevalence (% of frogs abnormal). Sample sites (N=462) from the 10-year survey are shown as circles; sites in significant hotspot clusters with high abnormality prevalence are indicated by a red circle outline. White polygons mask areas with high standard error (>0.023 prevalence units; Figure S9). Abnormality prevalence corresponded to latitude and longitude in a non-linear fashion (Figure S1, Table S4), as shown by solid lines (and dashed 95% confidence intervals) outside the map of the continental United States (Text S1). (A) Shows sites color coded and surface interpolated using the mean abnormality prevalence at each site. (B) Shows site color coding and surface interpolation using the minimum values at each site. (C) Shows site color coding and surface interpolation using the maximum values at each site.

**Figure 3:**
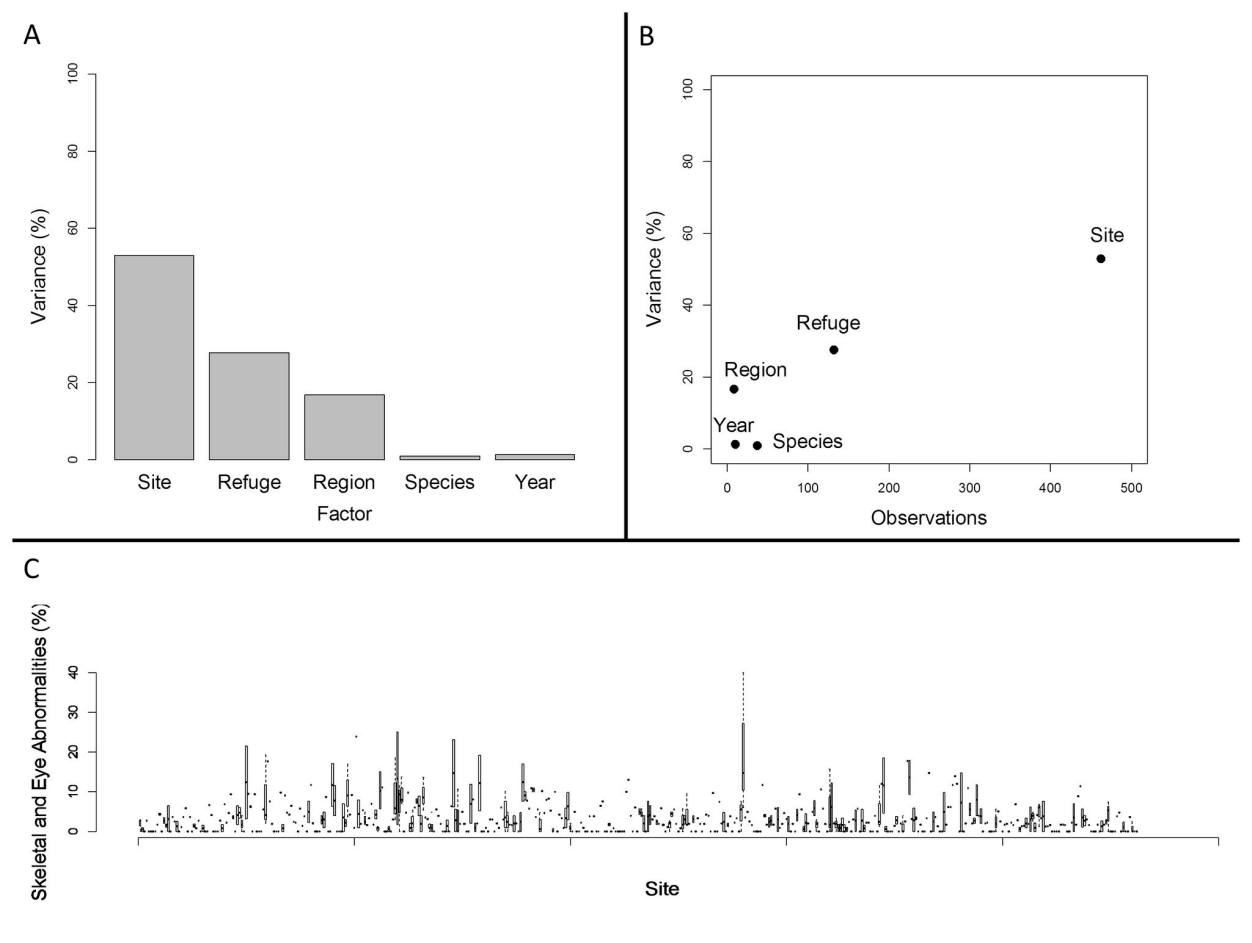
Variation in amphibian abnormalities. (A) Percentage of variance attributable to each factor tested in hierarchical models, which are shown as bars along the labeled X axis. Site is the individual wetland, Refuge is the USFWS Refuge, and Region is the USFWS Region (Figure S12). (B) Shows the same variance estimates plotted against the number of observations for each factor. (C) Box plots showing variation in abnormality prevalence during repeated sampling at individual sites (N=462).

The spatial distribution of abnormalities was strongly non-uniform (Figure 2, Figures S1–S9 for error and regional inset maps) and we identified discrete structure at several spatial scales. We discovered continental-scale, non-linear patterns in abnormality frequency as a function of both latitude and longitude using generalized additive mixed models (Figure 2A, Figure S10, Table S4). Factors associated with space at different scales collectively accounted for >97% of the variation in abnormalities (from variance partitioning analysis), with only minor contributions by amphibian species or year sampled (Figure 3). The proportion of variance explained decreased with spatial extent, and hence the site-level term explained a greater proportion of variance (53%) relative to refuge (28%) or region (17%, Figure 3). Overall, abnormality frequency was more similar than expected at scales of tens to hundreds of kilometers, but frequencies at sites close to one-another (~5 km, Figure S11 and Text S1) were less similar than expected by chance alone. 

We detected clusters of high-abnormality sites in the Mississippi River Valley (northeast Missouri, Arkansas, and northern Louisiana), throughout California, and in south-central and eastern Alaska using Getis-Ord [43] hotspot analyses (Figure 2, Figures S1-S9, and Text S1). Within these clusters, abnormality frequency often exceeded the national average of 2%, affecting up to 40% of emerging amphibians. The interpolated surface of expected abnormalities across the U.S. shows areas in Missouri, southern Arkansas, northern Louisiana, California, and Alaska as candidates for targeted subsequent investigations, and areas in the west-central U.S. where abnormalities were less common.

### Temporal and species variation in abnormality frequencies

**Figure 4:**
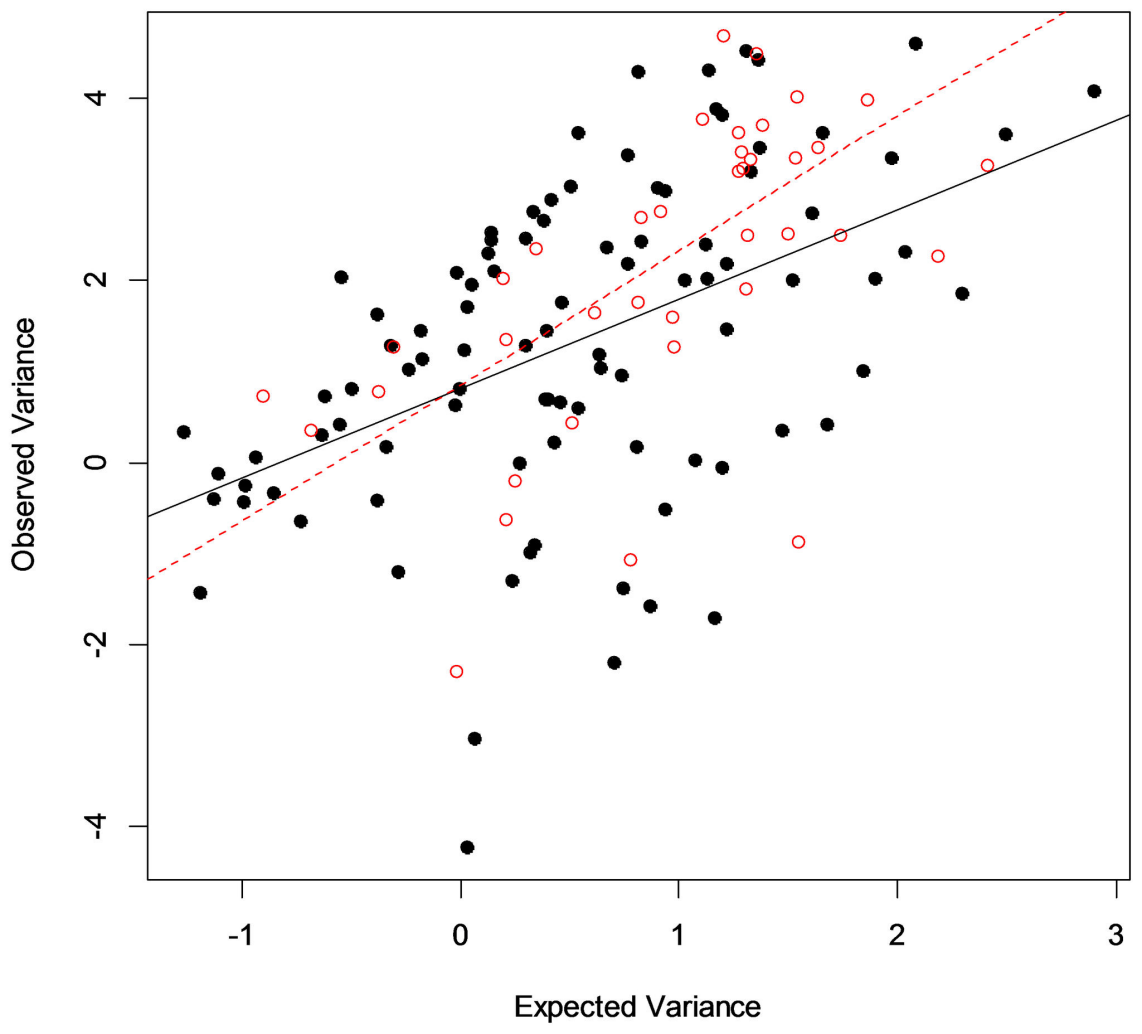
Differences in temporal variation between normal sites and hotspot clusters, measured by Taylor’s power law analysis. Sites in hotspot clusters (Text S1) are coded with open red circles, non-hotspot sites are coded with black filled circles. The dashed red line is the regression of expected versus observed site-level variance for hotspot cluster sites following the equation ln(V_obs_)=ln(*A*)+*b*ln(V_bin_), where the expected variance was calculated with the following formula: V_bin_=[*np*(1-*p*)], where *p* is the mean proportion abnormal at a site, and *n* is the group size, or number of frogs sampled at each site (Text S1). Observed variance (V_obs_) was calculated from repeated site visits using standard methods for estimating sample variance. For sites in hotspot clusters, the regression parameter estimates were ln(*A*)=0.84±0.36 (SEM) and *b*=1.48±0.28 (SEM). The slope of this line was significantly greater than one (t=1.73, with 39 df, p=0.046), implying significant aggregation, i.e., temporal variation dependent on the mean abnormality prevalence (*p*). For non-hotspot sites (solid black regression line) the parameter estimates for this relationship were ln(*A*)=0.81±0.18 (SEM) and *b*=0.98±0.18 (SEM). This slope was not significantly different than one (t=0.096, with 92 df, p=0.462) implying random temporal variation.

We found no evidence of synchronous, year-to-year variation or consistent differences in abnormality prevalence among amphibian species (Figure 3, Tables 1 and S5, and Text S1), although asynchronous variation in abnormalities through time clearly occurred (Figure 3C). Analyses of temporal variation in abnormality frequency showed a strong mean-variance relationship (as expected; Figure 4), with a regression slope of greater than one indicating aggregation in abnormalities [40,44]. Importantly, however, the degree to which a site was within a hotspot cluster (as measured by the Gi* statistic, [43]) significantly moderated the relationship between the actual and predicted temporal variance (Gi*Score regression parameter estimate = 0.15 ± 0.07SE, t = 2.15, p = 0.03), showing sites within hotspot clusters to be more variable through time than sites outside of them. Separating wetlands based on whether they were in hotspot clusters (significant Gi* statistic [43]) or not, and performing modified Taylor’s power law regressions for each subset, revealed that sites within hotspot clusters exhibited significantly more temporal variation than other sites (Figure 4). Overall, these analyses indicate that temporal dynamics in hotspot and non-hotspot sites differ, while other factors, including space at different scales, species, latitude, longitude, and the number of times a site had been sampled, did not improve our explanation of temporal variation (Text S1). 

**Table 1:** Model comparison for factors potentially influencing skeletal and eye abnormalities.

***Fixed Effects***	***DF***	***AIC***	***Δ AIC***
**Region**	**9**	**1386**	**0**
**Refuge**	**133**	**1387**	**1**
Year+Region	18	1393	7
Region+Species	26	1403	17
Size+Region+Species	27	1404	18
Year+Region+Species	35	1410	24
Site	463	1426	40
None	2	1429	42
Species	19	1435	48
Year	11	1437	51

## Discussion

Our analyses indicate that severely malformed frogs, such as those that originally prompted concern and controversy, were relatively rare across U.S. National Wildlife Refuges between 2000 and 2009. This study is the largest U.S. survey of abnormal amphibians to date, including examinations of 62,947 amphibians across the continental U.S., including Alaska. Analysis of the core dataset of 48,081 animals showed that the overall frequency of abnormal amphibians averaged just 2%, with roughly one-third of collections yielding no abnormal individuals (Table S2). This data set provides a robust volume of baseline data for characterizing the expected frequencies and types of abnormalities in wetland-breeding amphibians, previously the subject of repeated discussion and debate [4,17,18,23-25,30]. Correspondingly, it facilitates identification of sites or collections that are unusual in terms of either the frequency or composition of abnormalities and therefore requiring further investigation. These data will facilitate design of future studies at appropriate spatial and temporal scales.

Sites with an abnormality frequency >5% have previously been considered hotspots [2-4]. Most collections in our survey were below this 5% threshold, supporting earlier estimates of 0–2% for baseline abnormality frequencies, when sample sizes are adequate (see 2-4). The median abnormality prevalence in our survey was 2%, whereas collections with more than 5% abnormal frogs or toads were much less common (above the 75^th^ percentile, Table S2). Despite the low frequency of abnormal amphibians on average, we detected substantial variation in this pattern, with >150 collections falling in the tail of the frequency distribution (with between 5 and 40% abnormal; Table S2). 

These instances of high abnormality frequency often occurred in clusters of high abnormality hotspots with distinctively different dynamics than wetlands elsewhere. Our spatially explicit analyses showed trends in abnormalities along latitude and longitude that are largely explained by the locations of spatial clusters where abnormalities are high (Figure 2, Figures S1–S10, Table S4). Abnormalities in hotspot clusters (defined by our Getis-Ord [43] analysis, *Methods*) occurred at higher frequencies than other sites (hotspot median=4.0%, Interquartile (IQ) Range=1.5–8.5%; other median=1.9%, IQ Range=0.0–3.8%) and were spatially persistent at scales of tens to hundreds of kilometers. Interestingly, however, sites within hotspot clusters were also temporally dynamic (Figure 4), raising the question of whether they are driven by more variable environmental factors or have more variable intrinsic dynamics, consistent with several of the hypotheses for abnormalities that have been proposed (Table S1). 

Our hierarchical modeling results suggest two key interpretations regarding previously proposed hypotheses. First, species do not appear to be differentially vulnerable to abnormalities at the national scale despite large sample sizes (although species may vary in their vulnerability to different abnormality-causing agents at more local scales [26]). Second, asynchronous patterns of inter-annual variation in abnormality occurrence did not support a hypothesis implicating broad-scale extrinsic factors that change annually (e.g., weather) as primary causes of abnormality occurrence. It is important to note, however, that despite its spatial extent, this study was not designed to explicitly compare abnormality patterns in particular species or genera within sites or over time (i.e., most wetlands were sampled for less than two years (mean=1.7 years per site) over the 10-year study period), precluding a comprehensive comparison of long-term temporal trends.

Overall, our spatial patterns fell into two distinct categories: Clustering of multiple hotspot sites in space, and isolated instances of high-abnormality prevalence at single sites (Figure 2, Figures S1–S9). These patterns could arise from several potential causal scenarios. First, abnormalities within regional hotspot clusters may all have a single causal etiology if that factor varies in abundance or concentration across time and space. For instance, differences in habitat across sites may influence local effects of single stressors responsible for the abnormalities, like parasites [45], predators [17,18], or concentrations of teratogens in water [46](Table S1). Alternatively, hotspot clusters may share a single regional contributing factor, which must combine with other more dynamic factors to produce abnormalities. For example, widespread contamination with chemical pollutants (as sometimes seen in agricultural systems) may vary in direct toxicity due to other co-factors such as temperature or UVB [47-50], or may alter amphibian behavior or immunity to make them more vulnerable to biotic factors such as predators or parasites (e.g. 4,33). This requirement for the co-occurrence of causal factors in space and time represents the “multiple stressor” hypotheses for abnormal frogs (Table S1). Finally, consistent with isolated instances of high abnormality prevalence, individual hotspots may involve different causal factors such that abnormalities represent a series of discrete phenomena. For example, high abnormality frequencies might occur in wetlands that have localized contamination, such as historical landfills, coal spoils, or other waste disposal areas (e.g., [20,34,35,51-53], wetlands subjected to localized road or agricultural runoff [31,54], or those with conditions favorable to a high abundance of predators or parasites [14,55]. 

Given that our study focused on non-randomly selected sites located only on lands managed for wildlife conservation, an important question is the degree to which the current data are similar to or different from previous surveys of amphibian abnormalities. All sampling in the current study occurred within the USFWS Refuge system, which includes Refuges, Wetland Management Districts (WMDs), and Waterfowl Production Areas (WPAs), collectively comprising over 145 million acres. Thus, the first question we explore is whether hotspots identified in the current survey match in location and form to those reported in other, more regionally focused studies. Our analyses revealed hotspot clusters in previously identified areas (e.g. California [14,56,57] and Alaska [31,32]) and detected new hotspot clusters that require further research. The hotspot cluster in the lower Mississippi River Valley has received little attention as yet (but see 7, which documents increasing abnormality frequencies in this vicinity since the 1950s). We did not identify significant hotspot clusters where other investigators have reported hotspots in Minnesota [2] or Vermont [54], yet our field results are consistent with those prior findings. We only sampled four wetlands in Vermont, at the only two National Wildlife Refuges in this state (Figure 2, Figure S5). None of these wetlands were classified as being within a significant hotspot cluster by the Getis-Ord analysis [43], despite the fact that three of the four met the ≥5% abnormal hotspot criteria in at least one sampling event. The likely reason for a lack of significant clustering in this case is that high within-site variation reduced the average abnormality prevalence at each site, which influenced the Getis-Ord [43] results (median abnormality prevalence=2.0% for VT sampling, Text S1). Similarly, in Minnesota we sampled 4,328 animals from five Refuges, and seven sites in four Refuges had ≥5% abnormal amphibians in at least one sampling event (median abnormality prevalence=2.2% for MN). Yet, in Minnesota, we again found high within-site temporal variation, which resulted in a low mean abnormality prevalence, and a consequent lack of spatial clustering of high abnormality sites in this area (Text S1). 

Interestingly, however, even in areas where there was geographic congruence in identification of hotspots between the current study and prior reports, some differences are apparent in terms of the hotspot characteristics. California, for instance, which has repeatedly been reported as a malformation hotspot [14,16,56,58-60], was identified as a hotspot cluster in our Getis-Ord [43] analysis. In California we sampled 4,415 animals from 15 Refuges, with 17 of 31 sites having ≥5% abnormal amphibians in at least one collection (median abnormality prevalence=4.6%, mean=5.4%, max=23.2% for CA collections). However, prior sampling of off-refuge lands suggested higher frequencies of more severe malformations. An examination of 24,215 emerging amphibians from California resulted in an average (±1 SE) of 6.17 ± 0.7% abnormal, with up to 88% of individuals abnormal at a single wetland [14]. Although sites were selected through different criteria than the current study, the higher mean, and especially the higher range in abnormality frequencies, suggests that differences may exist in either the types of wetlands or how they are managed, at least in this region. 

Despite the fact that National Wildlife Refuges are managed “to conserve, protect, and enhance fish, wildlife, plants, and their habitats for the continuing benefit of the American people” (USFWS Mission Statement), it is important to clarify that Refuges across America harbor considerable variation in habitat and water quality. While some Refuges represent nearly undisturbed natural habitats, others have been affected by land uses that have led to habitat degradation within Refuge bounds. For example, the source of surface water managed by numerous Refuges is primarily agricultural drainage. Former military lands with residual contamination have been given to the USFWS to manage for wildlife once military activities cease. Due to pollution sources such as agriculture, mining, urban development, and atmospheric deposition, one-third of Refuges (178 Refuges and 17 WPAs) include one or more impaired water body (Clean Water Act (CWA) Section 303(d) impaired waters, [61]). Moreover, the USFWS does not always own mineral rights underlying Refuges, and this has led to development of oil and gas resources on roughly one-quarter of Refuges (155 of 575 Refuges in 2003, [62]). Some Refuges represent the best wild habitats left in an area, yet others are managed for wildlife despite the conservation challenges presented by past, present, and current adjacent land use. 

Finally, it is important to note that this study was not designed to measure status or trends of amphibian populations. Therefore, use of the number of amphibians in our collections to infer trends about population status or persistence would be inappropriate. Our protocols directed field crews to sample between 50 and 100 metamorphosing amphibians of a single species during each collection event and determine the proportion of these that were abnormal. The total sampling effort required to collect this number of amphibians varied across sites, and therefore does not reflect amphibian abundance. Sample size of individual collections may have varied due to timing of the collection relative to amphibian metamorphosis, difficult sampling conditions, the collection of non-target species (as per our protocols), or reduced effort due to unrelated logistics. Research conducted over the same period as our study documents higher than anticipated rates of amphibian decline in numerous U.S. amphibian populations, including in species that are currently considered of little conservation concern [15]. Other studies have found abnormalities to affect individual fitness [60], but effects on population dynamics need further research. Anecdotally, it was not uncommon for investigators in this study to record a high prevalence of abnormal amphibians in one collection event, yet be subsequently unable to collect an adequate sample size for abnormality assessments during subsequent site visits. This pattern has been documented in prior investigations [2,8] and occurred often enough in this study that most regional coordinators could present several such examples. 

## Conclusions

Results of multiple analyses of this large-scale data set suggest that factors at local to regional scales likely drive abnormalities. The pattern of regional hotspot clusters combined with high site-to-site variation is consistent with either single causative factors that vary spatially and temporally or multiple factors that co-occur in time to produce high abnormality frequencies. The local to regional spatial scale of amphibian abnormality occurrence is encouraging from the perspectives of amphibian conservation and wetland management because it suggests that abnormalities can be reduced by an improved understanding of their causative factors, which occur at a manageable scale. These findings refute several hypotheses for amphibian abnormalities (null, species, and climate, Table S1), and direct future investigations to focus on factors contributing to hotspot clusters. We suggest that findings of high percentages of abnormal frogs, particularly those that cluster together spatially, warrant targeted investigation of the local factors causing abnormalities and their consequences for the sustainability of affected amphibian populations. 

## Materials and Methods

### Basis for project

In 2000, the U.S. Congress asked agencies within the Department of the Interior (the USFWS, the U.S. Geological Survey, the National Park Service, and the Bureau of Land Management) to address growing concerns about the health of amphibians and their populations. In response to this request, the USFWS Division of Environmental Quality began the Abnormal Amphibian Monitoring Program (AAMP). The aim of AAMP was to characterize the geographic distribution of amphibian abnormalities on National Wildlife Refuge lands.

### Site and species selection

We selected areas within the Refuge system for sampling based on criteria including: 1) presence of frogs and toads (amphibians); 2) availability of sites with suitable and accessible habitat; and 3) availability of Refuge staff, especially for the monitoring of tadpole development. We focused solely on frogs and toads for this survey. When a Refuge had frogs of the *Ranidae* (true frog) family, we prioritized their collection because of considerable prior documentation of abnormalities in *Rana* and *Lithobates* species within this family [3], and the family’s wide distribution across the United States. We used the taxonomy of Crother [63], which reclassified many frogs formerly of the genus *Rana* as *Lithobates* and toads formerly of the genus *Bufo* as *Anaxyrus*. We ultimately sampled 32 species, with five additional taxa only identified to genus (Table S5). We identified potential amphibian breeding areas based on site visits and discussions with Refuge personnel and local experts. All sites were within Refuge, Wetland Management District (WMD), or Waterfowl Production Area (WPA) boundaries. Sites were usually small, isolated wetlands or other ephemeral water bodies, such as agricultural or roadside ditches. Refuge staff, interns, and/or volunteers monitored the development of tadpoles at each site and helped determine the timing of collections. 

### Field Methods

We evaluated and monitored sites according to Standard Operating Procedures (SOPs) developed by the Program [64] (also included with our data submission at Dryad Digital Repository, http://doi.org/10.5061/dryad.dc25r). For each selected Refuge, field crews tried to obtain a collection from at least two sites, where they examined amphibians at developmental stages from forelimb emergence through full tail resorption. We assessed malformation frequency only in metamorphs ([65], stages 42–46), the life-history stage between larva and juvenile, to control for variation in abnormality prevalence as a result of developmental stage [31]. Adults were not sampled because severely abnormal animals are less likely to survive to adulthood [13] and are more difficult to capture *en masse*. Our goal was to obtain at least 50 metamorphs of a species at a site to provide confidence in our calculation of abnormality prevalence. We attempted to repeat the sampling of a Refuge within two to three years of the initial survey. We held the captured amphibians in containers with site water and vegetation until examination. We examined all individuals for external abnormalities and measured snout to vent length, tail length, and developmental stage according to SOPs ([64]; Text S1). 

### Ethics Statement

Collection, handling, and holding procedures were humane and consistent with American Society of Ichthyologists and Herpetologists guidelines [66]. No invasive procedures were conducted on live animals. A subset of animals was sent for further diagnostic evaluation using radiography; these were euthanized by overdose of tricaine methanesulfonate (MS-222; Argent Chemical Laboratories, Redmond, WA).

### Description of the data set

*Total amphibians examined in the field*: The field data were organized by collection, where a collection is a sampling of one or more individuals of a species of amphibian at a site. Altogether we conducted a total 1,477 collections including 68,359 individual amphibians. *Description of the core dataset for spatially implicit and explicit models*: Approximately a quarter of these collections (397 collections or 27%) had ≤10 individuals, but over half (878 collections or 59%) had ≥30. Preliminary analyses showed that estimates of abnormality prevalence were highly variable for collections that included <30 individuals. We included collections with a minimum sample size of 30 individuals in the *core dataset* for analysis, which retained 92% of animals sampled (62,947). Our emphasis was on obtaining a broad spatial sample, however, field protocols [63] specified that if a site exceeded 3% skeletal and/or eye abnormalities in two of the first three years of sampling, then that site qualified for additional sampling to assess temporal variation in abnormality prevalence. To prevent overweighting of these high abnormality sites, only the first two years of data were retained in the *core dataset*. Any additional collection events after two years of sampling were excluded from all analyses presented in this paper except the power law analysis that dealt explicitly with temporal variation and the kriging for the minimum and maximum prevalence panel maps in Figure 2. If sites had more than two years of sampling but did not exceed 3% abnormality prevalence in the first two years, then all years were arbitrarily retained in the *core dataset* for analysis (this additional sampling occurred for 18 sites and 30 collection events, and represented 2,592 individual amphibians). Thus, the core dataset consisted of 48,081 amphibians sampled during 675 collections at 462 sites located in 132 Refuges. *Description of the data set for Power Law Analysis*: Because this analysis focused on temporal variation, we used a data set that differed from the *core dataset*. We began by including all collections with ≥30 individuals, from all sites that had been sampled two or more times in different years and where abnormal amphibians occurred in at least some collections. This resulted in a data set including 30,020 frogs from 479 collections at 136 sites within 60 Refuges. All of the data used in this paper, in addition to other data collected by the program, our field forms, and our SOPs have been posted to Data Dryad (http://doi.org/10.5061/dryad.dc25r).

### Accounting for spatial and temporal autocorrelation in spatial and hierarchical models

We specified the most simple random effects structure possible to deal with spatial and temporal autocorrelation in the spatially implicit and explicit models [67]. We compared various combinations of the following factors as nested and non-nested random effects in models with no fixed effects: USFWS Region, Refuge, Site, Year, and Species (Table S6). The best model (judged by Akaike’s Information Criterion (AIC)) included site, year, and species as a single, concatenated random effect (“site.year.species”). Nevertheless, the difference in AIC between this more complex model and a simpler model with a site and year concatenated variable (“site.year”) was only five points (Table S6), showing the more complex model to be only a slightly better relative fit than the simpler one. On this basis and the overall lack of sites with adequate sampling of multiple, concurrent amphibian species, we elected to use the simpler model. We used this “site.year” random effect to account for correlation among collections introduced by our field sampling process in all hierarchical models and some spatial models (see methods for the individual models for details). 

### Spatially explicit analyses

The sampling unit for spatial analyses was the individual collection, except for kriging and the Getis-Ord analyses, which averaged at the site level (Figure 2). *Generalized additive mixed models*: To evaluate non-linear patterns along longitude and latitude, we fit generalized additive mixed models (GAMMs) to skeletal and eye abnormality prevalence (mgcv library in R [67]). We estimated parameters using penalized quasi-likelihood (1,000 iterations), and fit models with a logit link function using a binomial error distribution. We calculated equivalent degrees of freedom (edf) to determine whether patterns were linear (edf=1) or significantly non-linear (edf>1). We included a concatenated site.year variable as a random effect to account for autocorrelation at sites (see *Accounting for spatial and temporal autocorrelation in spatial and hierarchical models* section above). *Identification of significant hotspot clusters*: We used Getis-Ord analyses [42] in ArcGIS [68] to evaluate significant clustering of high-abnormality sites (i.e., hotspot clusters) using abnormalities averaged within sites across collections. Gi* values with a z-score (i.e. standard deviation) >1.96 were considered significantly clustered (Text S1)*. Lag matrix models to assess the scale of spatial autocorrelation*: We evaluated at what spatial scale abnormality frequencies were similar by creating a lag-matrix model using multiple regression on distance matrices [69,70] with sample year and distance between sites as predictors and (ln) abnormality prevalence per collection as the response (Text S1). *Prediction of abnormality frequencies between sample locations*: To supplement our spatially explicit analyses of hotspot clusters (above) and visualize predicted trends across the U.S., we created a predictive surface of skeletal and eye abnormality prevalence by interpolating between abnormalities averaged across collections at sample sites using ordinary kriging with exact measurement in ArcGIS 10.0 (Figure 2, Figures S1–S9) [68]. We used an iterative cross-validation technique to optimize models and chose the best model from multiple iterations as the one with the lowest average standard error and with a root-mean square standardized error closest to one. One site in Alaska was deleted from this analysis because it was identified in a prior study as an outlier [30], causing a prediction of high abnormality frequencies in a relatively data-poor area. Because some areas of the country were sparsely sampled, we masked areas with high standard error (>0.023 prevalence units—upper quartile). This analysis was intended to predict spatial trends across the U.S. using data collected at wildlife refuges, and does not incorporate any predictors (e.g., climate, land use) for the observed spatial structure. We recognize that data at wildlife refuges may not represent expected abnormalities off refuges, but our surface should serve as a baseline prediction and provide information for targeting future surveys. 

### Spatially implicit hierarchical modeling

We used Generalized Linear Mixed Effects Regression (GLMM) models in R ([67], library lme4) to estimate a mean national abnormality frequency and to compare species, time, and space as predictors of collection level abnormality prevalence (Text S1). Our models used a logit link function, weighted for sample size of each collection event, and assumed a binomial error distribution. We used AIC to compare model fit of individual and combined factors that tested for effects of space, time, and species on abnormality frequency. All of these models except the simple variance components analysis used the concatenated (site.year) random effects structure we determined to be most appropriate in the optimization step described above (see *Accounting for spatial and temporal autocorrelation* section). The sampling unit for these analyses was the individual collection event, and all hierarchical analyses used only the *core dataset* (Text S1). 

### Power law analysis

We used the binomial form of Taylor’s Power Law [39] to test for aggregation in our data set (Text S1). This analysis tests the strength of a linear regression relationship between the calculated site-level variance and the variance predicted from randomly distributed data following a binomial distribution. Once we had performed this regression, we asked whether the variation we found was predictable by the following factors or variables by adding them one at a time as predictors into the linear regression model: Space (Region, State, or Refuge), time (calendar year sampled as a factor variable, or number of collection events as a continuous variable), species (coded into genus groupings for species with fewer than 500 individuals sampled, as above), and the Getis Gi* statistic [42], which provided a continuous variable measuring the degree to which sites with low abnormality prevalence (low and negative Gi* score) or high abnormality prevalence (high and positive Gi* score) cluster spatially. Of all these variables tested, the Getis Gi* statistic was the only significant predictor for the observed variance (V_obs_). Therefore, we separated the sites into two groups based on whether they were within a significant hotspot cluster in Getis-Ord analyses [42] or not, and tested the Taylor’s Power Law relationship for hotspot and non-hotspot groups to obtain separate estimates of slope (b) and intercept (ln(*A*)) (Figure 4). 

## Supporting Information

Figure S1 **Geographic distribution of amphibian abnormalities with hotspot clusters in the Pacific Northwest (USFWS Region 1).** Shows sites color coded and surface interpolated using the mean abnormality prevalence at each site. Warmer colors represent higher predicted abnormality prevalence (% of frogs abnormal). Sample sites from the 10-year survey are shown as circles; sites in significant hotspot clusters with high abnormality prevalence are indicated by a red circle outline. White polygons mask areas with high standard error (>0.023 prevalence units; Figure S9).
(TIF)

Figure S2 **Geographic distribution of amphibian abnormalities with hotspot clusters in the Desert Southwest (USFWS Region 2).** Shows sites color coded and surface interpolated using the mean abnormality prevalence at each site. Warmer colors represent higher predicted abnormality prevalence (% of frogs abnormal). Sample sites from the 10-year survey are shown as circles; sites in significant hotspot clusters with high abnormality prevalence are indicated by a red circle outline. White polygons mask areas with high standard error (>0.023 prevalence units; Figure S9).
(TIF)

Figure S3 **Geographic distribution of amphibian abnormalities with hotspot clusters in the Upper Midwest (USFWS Region 3).** Shows sites color coded and surface interpolated using the mean abnormality prevalence at each site. Warmer colors represent higher predicted abnormality prevalence (% of frogs abnormal). Sample sites from the 10-year survey are shown as circles; sites in significant hotspot clusters with high abnormality prevalence are indicated by a red circle outline. White polygons mask areas with high standard error (>0.023 prevalence units; Figure S9).
(TIF)

Figure S4 **Geographic distribution of amphibian abnormalities with hotspot clusters in the Southeast (USFWS Region 4).** Shows sites color coded and surface interpolated using the mean abnormality prevalence at each site. Warmer colors represent higher predicted abnormality prevalence (% of frogs abnormal). Sample sites from the 10-year survey are shown as circles; sites in significant hotspot clusters with high abnormality prevalence are indicated by a red circle outline. White polygons mask areas with high standard error (>0.023 prevalence units; Figure S9).
(TIF)

Figure S5 **Geographic distribution of amphibian abnormalities with hotspot clusters in the Northeast (USFWS Region 5).** Shows sites color coded and surface interpolated using the mean abnormality prevalence at each site. Warmer colors represent higher predicted abnormality prevalence (% of frogs abnormal). Sample sites from the 10-year survey are shown as circles; sites in significant hotspot clusters with high abnormality prevalence are indicated by a red circle outline. White polygons mask areas with high standard error (>0.023 prevalence units; Figure S9).
(TIF)

Figure S6 **Geographic distribution of amphibian abnormalities with hotspot clusters in the Mountain-Prairie region (USFWS Region 6).** Shows sites color coded and surface interpolated using the mean abnormality prevalence at each site. Warmer colors represent higher predicted abnormality prevalence (% of frogs abnormal). Sample sites from the 10-year survey are shown as circles; sites in significant hotspot clusters with high abnormality prevalence are indicated by a red circle outline. White polygons mask areas with high standard error (>0.023 prevalence units; Figure S9).
(TIF)

Figure S7 **Geographic distribution of amphibian abnormalities with hotspot clusters in Alaska (USFWS Region 7).** Shows sites color coded and surface interpolated using the mean abnormality prevalence at each site. Warmer colors represent higher predicted abnormality prevalence (% of frogs abnormal). Sample sites from the 10-year survey are shown as circles; sites in significant hotspot clusters with high abnormality prevalence are indicated by a red circle outline. White polygons mask areas with high standard error (>0.023 prevalence units; Figure S9).
(TIF)

Figure S8 **Geographic distribution of amphibian abnormalities with hotspot clusters in California and Nevada (USFWS Region 8).** Shows sites color coded and surface interpolated using the mean abnormality prevalence at each site. Warmer colors represent higher predicted abnormality prevalence (% of frogs abnormal). Sample sites from the 10-year survey are shown as circles; sites in significant hotspot clusters with high abnormality prevalence are indicated by a red circle outline. White polygons mask areas with high standard error (>0.023 prevalence units; Figure S9).
(TIF)

Figure S9 **Predicted standard error estimates from the kriging analysis using mean abnormality prevalence at each site.** Error is reported in prevalence units (0.01 prevalence unit=1%).
(TIF)

Figure S10 **Trends in skeletal and eye abnormalities by latitude (left) and longitude (right).** We detected significant non-linear trends in skeletal and eye abnormalities along latitude and longitude after accounting for temporal autocorrelation (Table S4). Peaks along trend lines correspond with spatial hotspots from Getis-Ord Gi* analyses that occur in Alaska, the central U.S. (Mississippi River Valley), and California (Figure 2). The solid smooth line is a trend estimated by local regression smoothing (LOESS) using generalized additive modeling and dashed lines represent 95% confidence intervals. The y axes represent the effect of latitude or longitude on skeletal and eye abnormality percentages at sites.
(DOCX)

Figure S11 **Lag matrix model representing distance classes (kilometers) on the x-axes and slope from regressions between inter-site distance and (log) difference in abnormality prevalence for each distance class on the y-axis.** Lag-matrix models did not detect a year effect, but significant positive relationships between geographic distance and (log) skeletal and eye abnormalities were detected at approximate inter-region, inter-site, and at one intermediate scale. Significant negative associations were detected at two small spatial scales (0–1 km and 1–5 km). Significant regressions are indicated by filled black symbols. Mean distance between sites within Refuges (Site) was 63 km, between Refuge centroids within regions (Refuge) was 512 km, and between region centroids (Region) was 4,752 km.
(DOCX)

Figure S12 **USFWS regions.** The USFWS has divided the country into eight administrative regions, shown in this figure. These Regions were used to test for coarse-scale spatial differences in abnormality frequency in the hierarchical models.
(DOCX)

Table S1 **Hypotheses for amphibian abnormalities (adapted from Johnson et al. 2010).** Includes a brief review of each current hypothesis for the causes of skeletal and eye abnormalities in amphibians and a literature cited section.
(DOCX)

Table S2 **Percentile ranks of skeletal and eye abnormalities in collection events in the *coredataset*** (n=675 Collections and 48,081 amphibians).
(DOCX)

Table S3 **Abnormalities found during field surveys.** Data presented are for the *core* *dataset*. Some individuals had more than one distinct abnormality. Bolded rows are summary data.
(DOCX)

Table S4 **Results from generalized additive mixed modeling of skeletal and eye abnormalities.** Edf: equivalent degrees of freedom. Edf values closer to 1 suggest a linear relationship, and larger edf values correspond to increasingly nonlinear relationships.
(DOCX)

Table S5 **Numbers of individuals of each amphibian species sampled in the *coredataset*.** On a national basis, we identified 32 amphibians to species. An additional 5 groupings summarize amphibians only to genus.
(DOCX)

Table S6 **Summary of random effects model structures evaluated.**
(DOCX)

Text S1 **Supplemental Methods and Discussion.** This file includes additional methodological descriptions for abnormality classification, data management, spatially explicit analyses, spatially implicit analyses, power law analyses, and additional discussion of the previously identified Minnesota and Vermont hotspots and how they relate to this dataset.
(DOCX)
