# Supplementary figures and images for: Localized Hotspots Drive Continental Geography of Abnormal Amphibians on U.S. Wildlife Refuges
Source: PLoS One. 2013 Nov 18;8(11):e77467. doi: 10.1371/journal.pone.0077467 (PMC3832516; doi:10.1371/journal.pone.0077467)

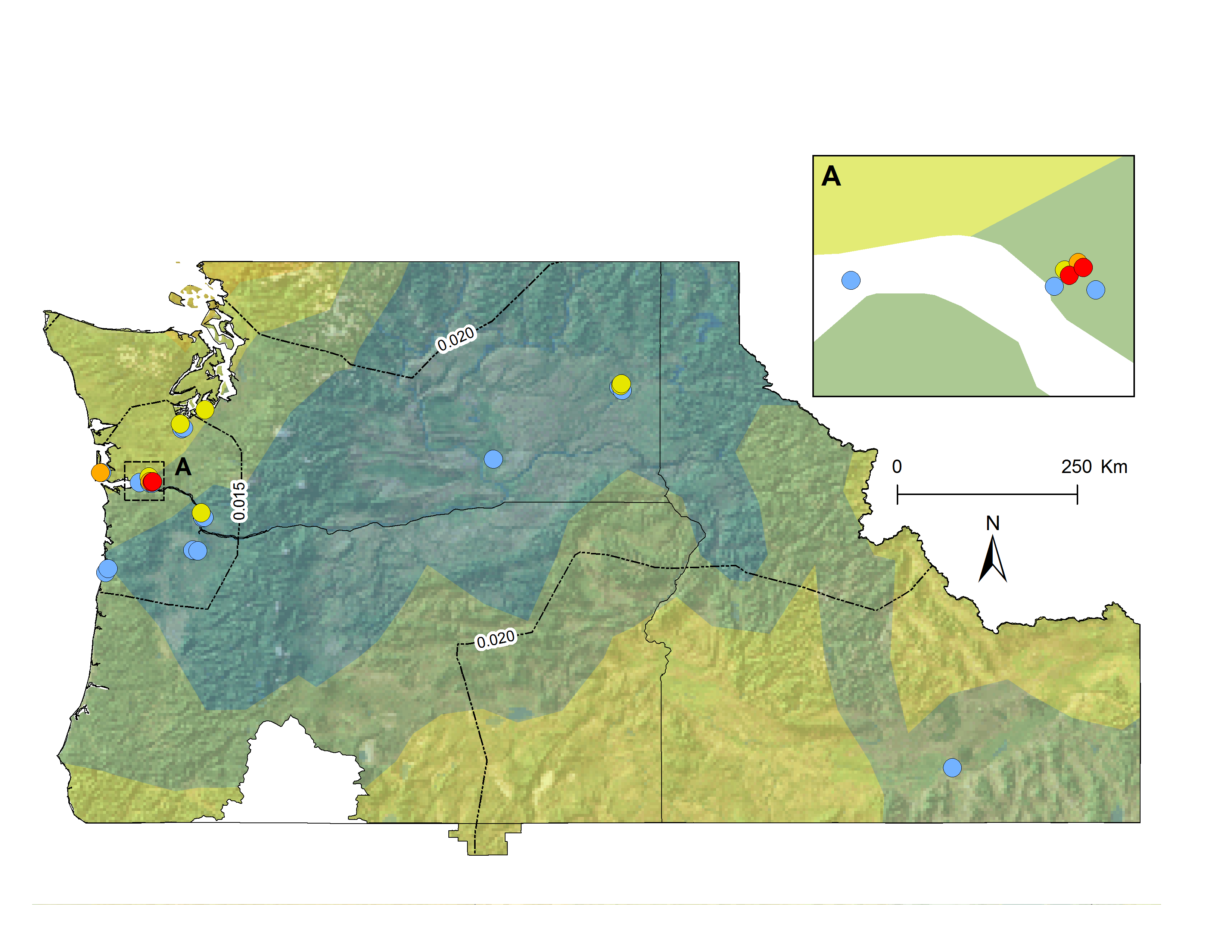

Supplement: Figure S1 — Geographic distribution of amphibian abnormalities with hotspot clusters in the Pacific Northwest (USFWS Region 1). Shows sites color coded and surface interpolated using the mean abnormality prevalence at each site. Warmer colors represent higher predicted abnormality prevalence (% of frogs abnormal). Sample sites from the 10-year survey are shown as circles; sites in significant hotspot clusters with high abnormality prevalence are indicated by a red circle outline. White polygons mask areas with high standard error (>0.023 prevalence units; Figure S9). (TIF) [file pone.0077467.s001.tif]

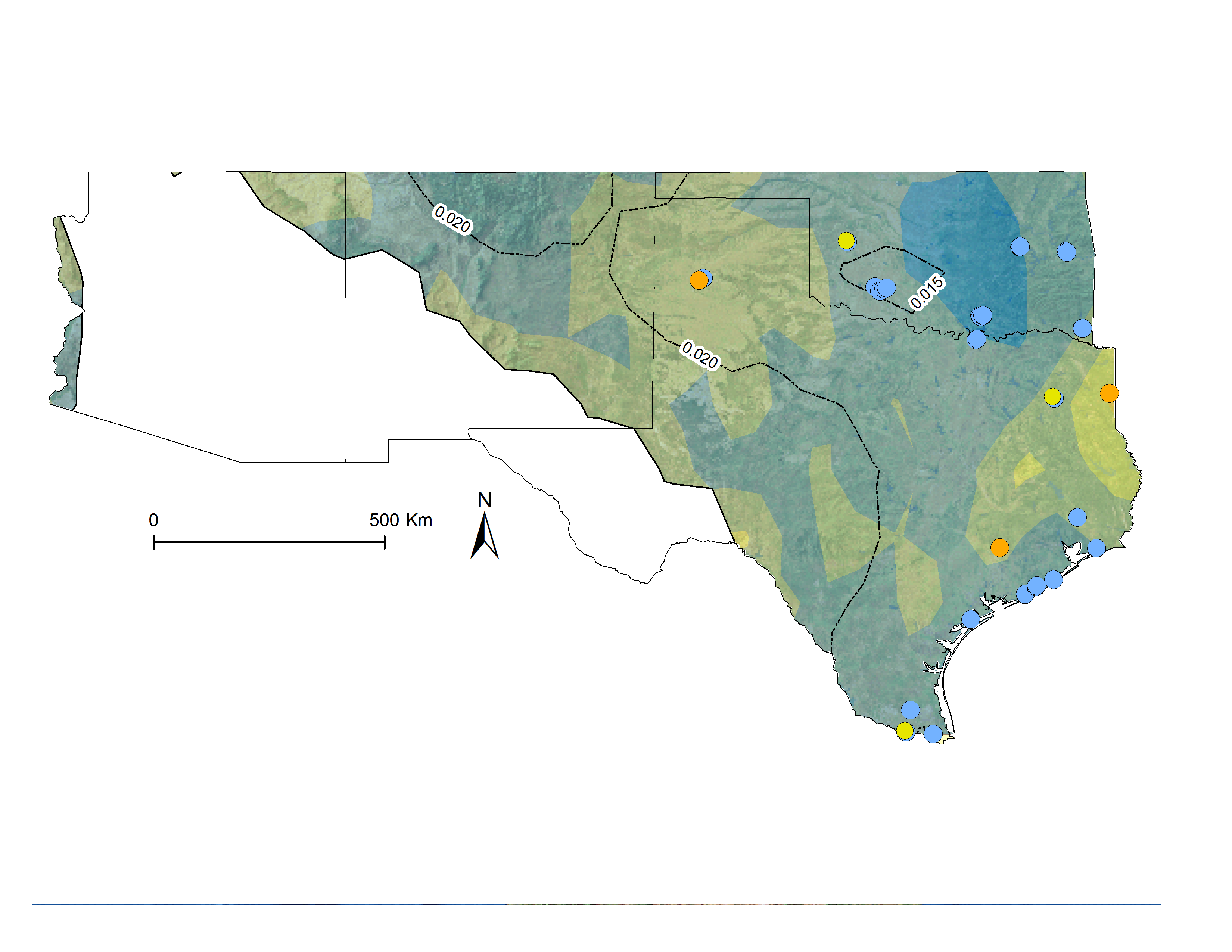

Supplement: Figure S2 — Geographic distribution of amphibian abnormalities with hotspot clusters in the Desert Southwest (USFWS Region 2). Shows sites color coded and surface interpolated using the mean abnormality prevalence at each site. Warmer colors represent higher predicted abnormality prevalence (% of frogs abnormal). Sample sites from the 10-year survey are shown as circles; sites in significant hotspot clusters with high abnormality prevalence are indicated by a red circle outline. White polygons mask areas with high standard error (>0.023 prevalence units; Figure S9). (TIF) [file pone.0077467.s002.tif]

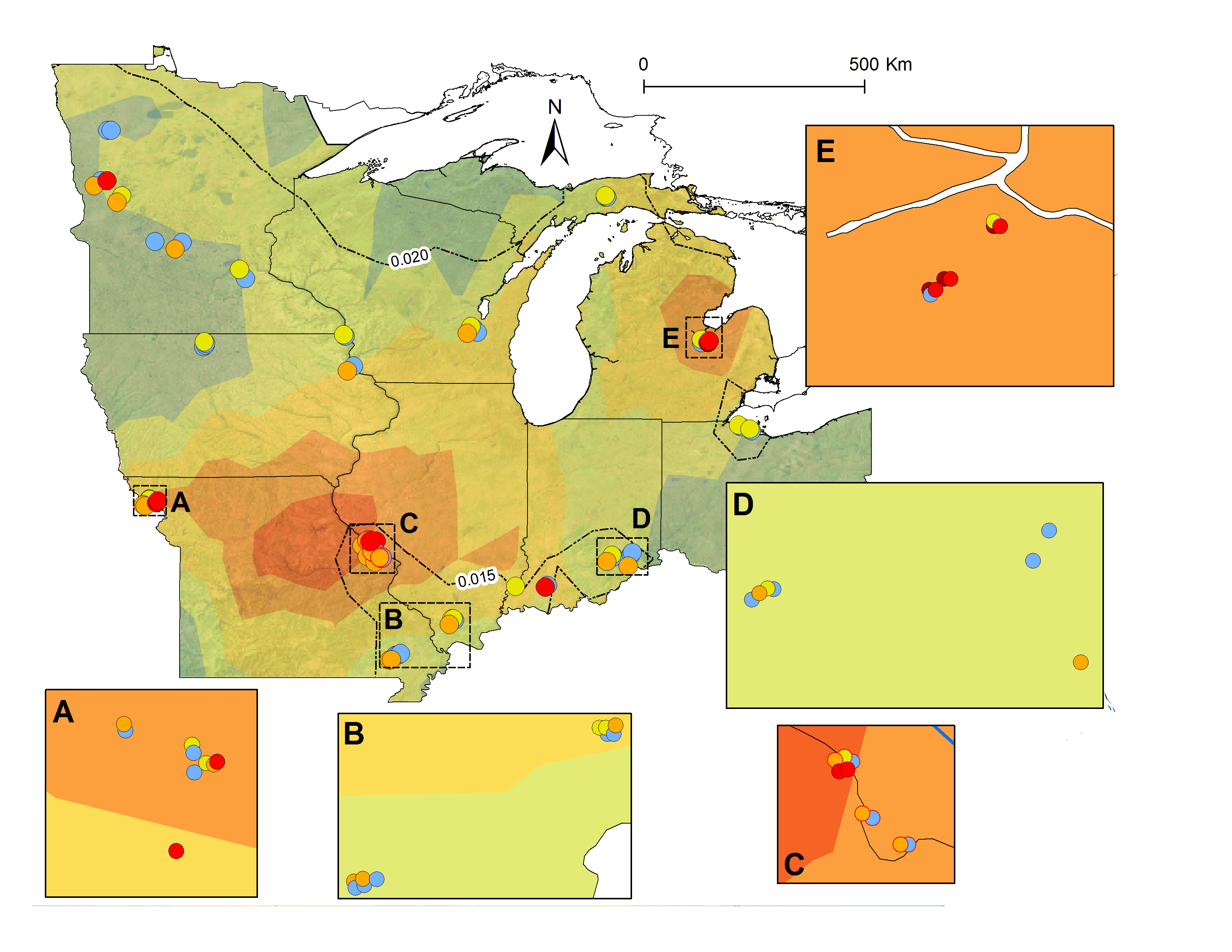

Supplement: Figure S3 — Geographic distribution of amphibian abnormalities with hotspot clusters in the Upper Midwest (USFWS Region 3). Shows sites color coded and surface interpolated using the mean abnormality prevalence at each site. Warmer colors represent higher predicted abnormality prevalence (% of frogs abnormal). Sample sites from the 10-year survey are shown as circles; sites in significant hotspot clusters with high abnormality prevalence are indicated by a red circle outline. White polygons mask areas with high standard error (>0.023 prevalence units; Figure S9). (TIF) [file pone.0077467.s003.tif]

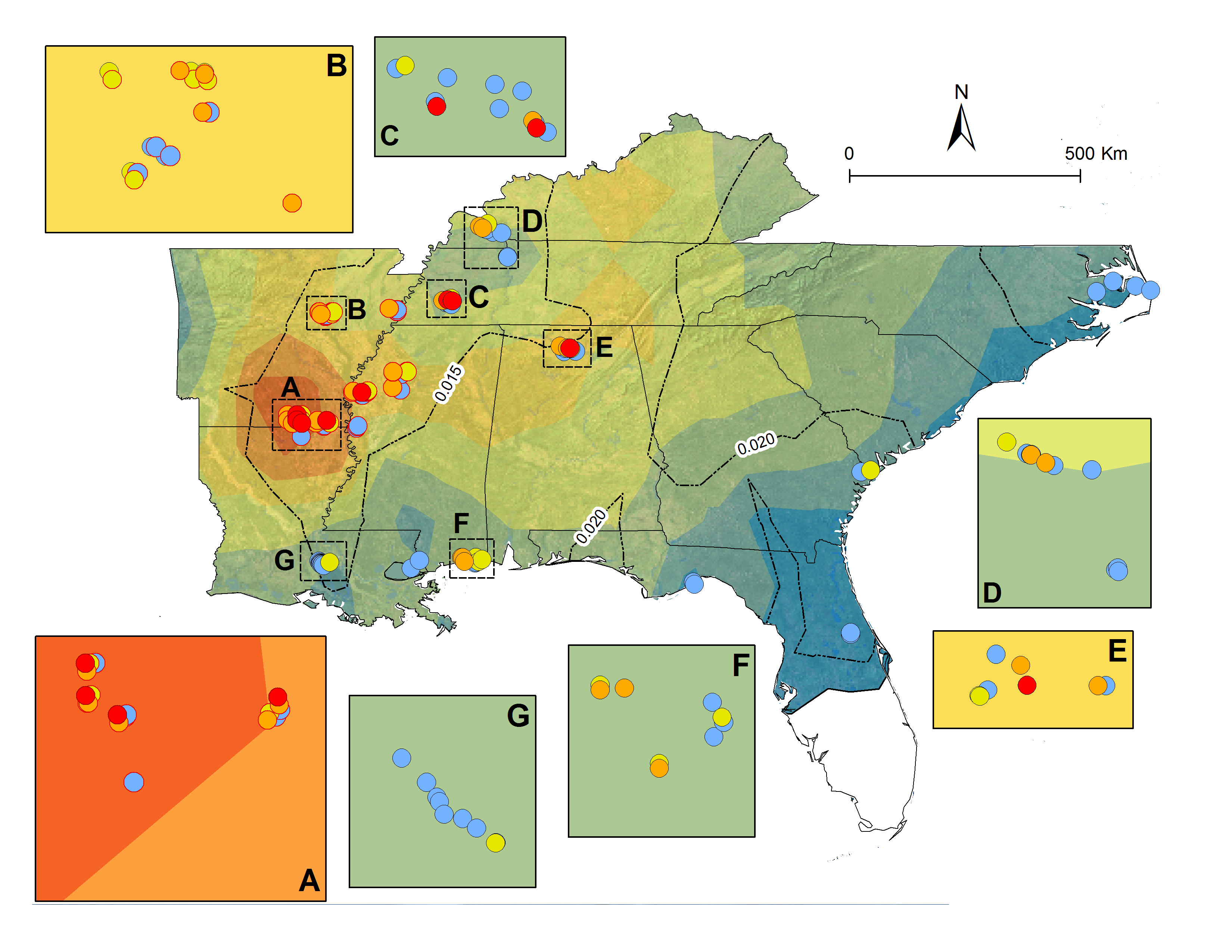

Supplement: Figure S4 — Geographic distribution of amphibian abnormalities with hotspot clusters in the Southeast (USFWS Region 4). Shows sites color coded and surface interpolated using the mean abnormality prevalence at each site. Warmer colors represent higher predicted abnormality prevalence (% of frogs abnormal). Sample sites from the 10-year survey are shown as circles; sites in significant hotspot clusters with high abnormality prevalence are indicated by a red circle outline. White polygons mask areas with high standard error (>0.023 prevalence units; Figure S9). (TIF) [file pone.0077467.s004.tif]

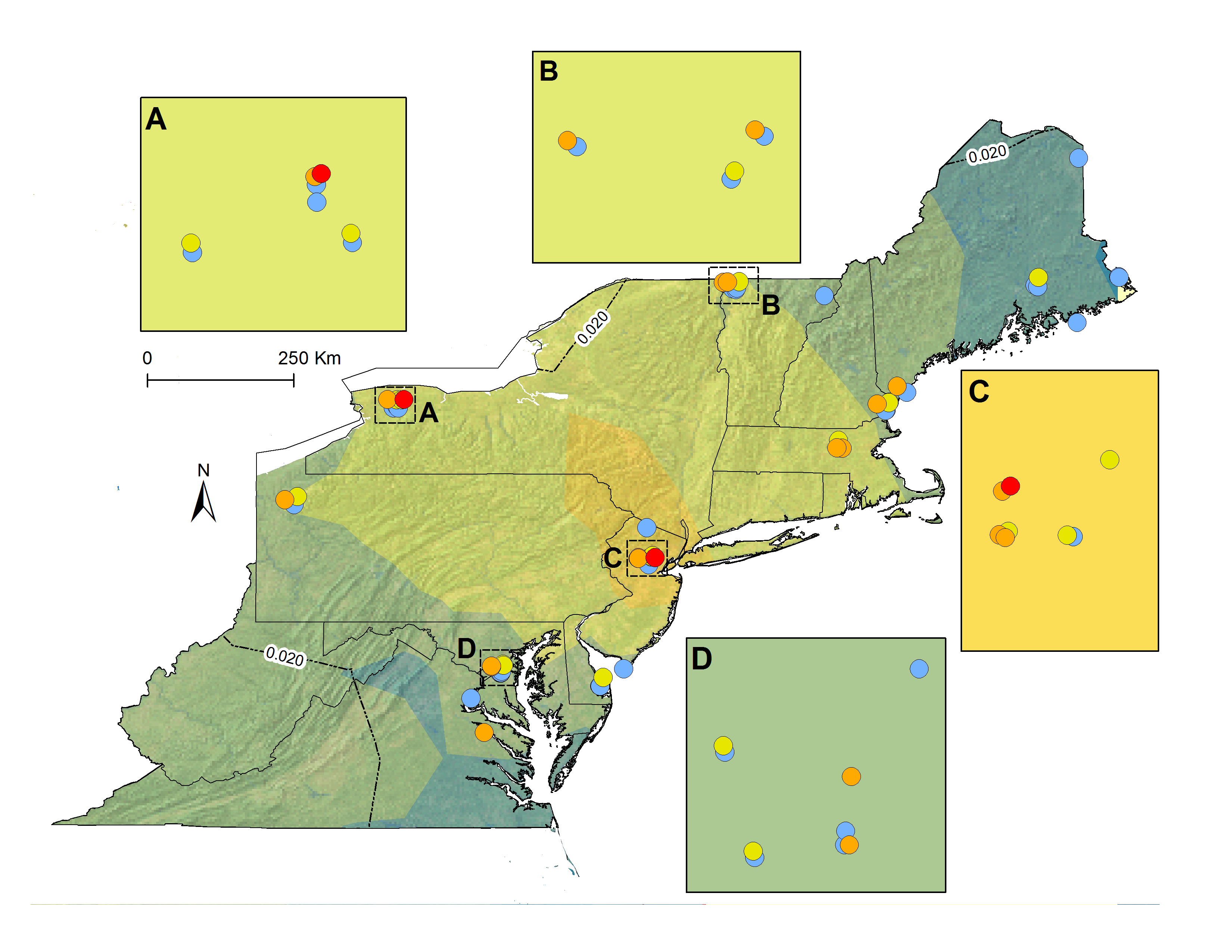

Supplement: Figure S5 — Geographic distribution of amphibian abnormalities with hotspot clusters in the Northeast (USFWS Region 5). Shows sites color coded and surface interpolated using the mean abnormality prevalence at each site. Warmer colors represent higher predicted abnormality prevalence (% of frogs abnormal). Sample sites from the 10-year survey are shown as circles; sites in significant hotspot clusters with high abnormality prevalence are indicated by a red circle outline. White polygons mask areas with high standard error (>0.023 prevalence units; Figure S9). (TIF) [file pone.0077467.s005.tif]

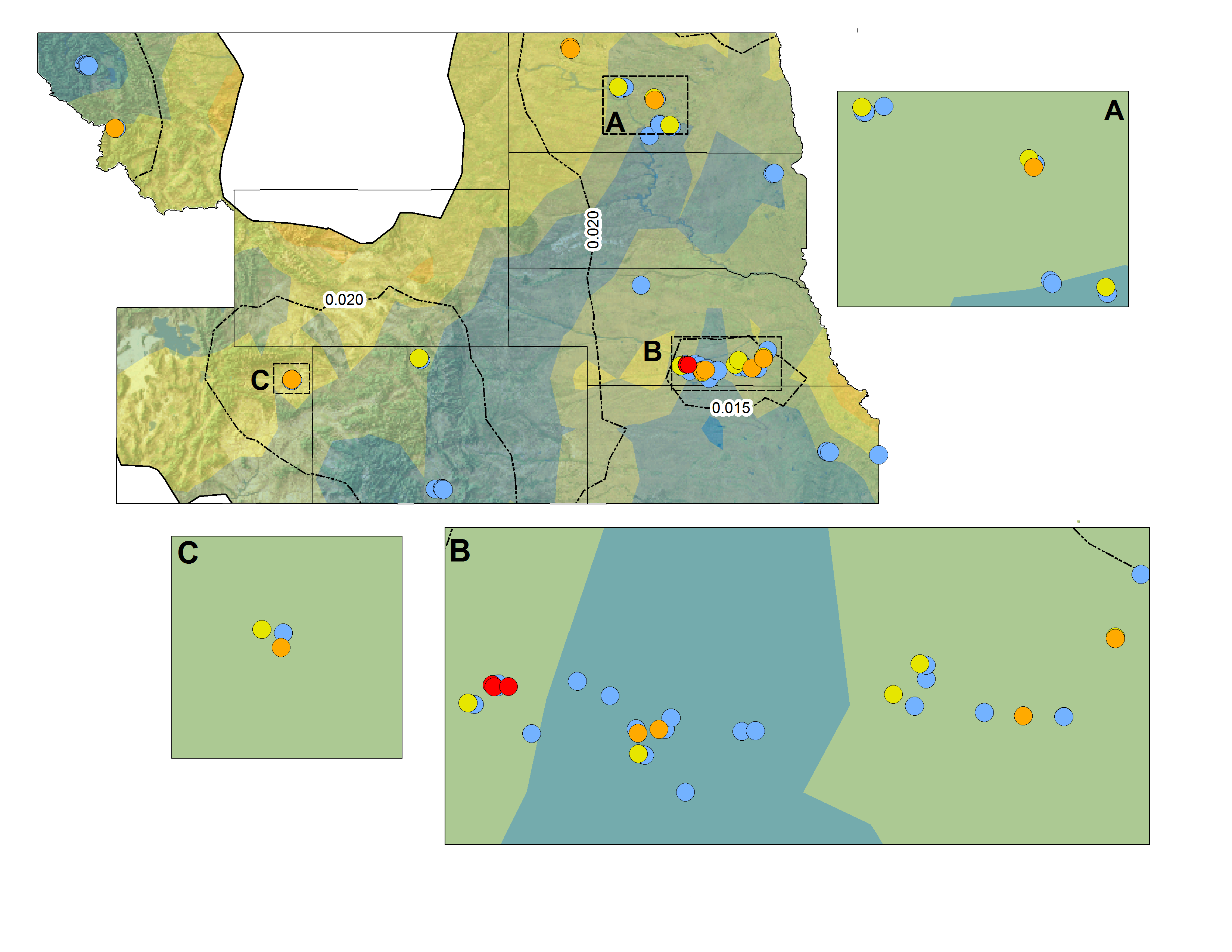

Supplement: Figure S6 — Geographic distribution of amphibian abnormalities with hotspot clusters in the Mountain-Prairie region (USFWS Region 6). Shows sites color coded and surface interpolated using the mean abnormality prevalence at each site. Warmer colors represent higher predicted abnormality prevalence (% of frogs abnormal). Sample sites from the 10-year survey are shown as circles; sites in significant hotspot clusters with high abnormality prevalence are indicated by a red circle outline. White polygons mask areas with high standard error (>0.023 prevalence units; Figure S9). (TIF) [file pone.0077467.s006.tif]

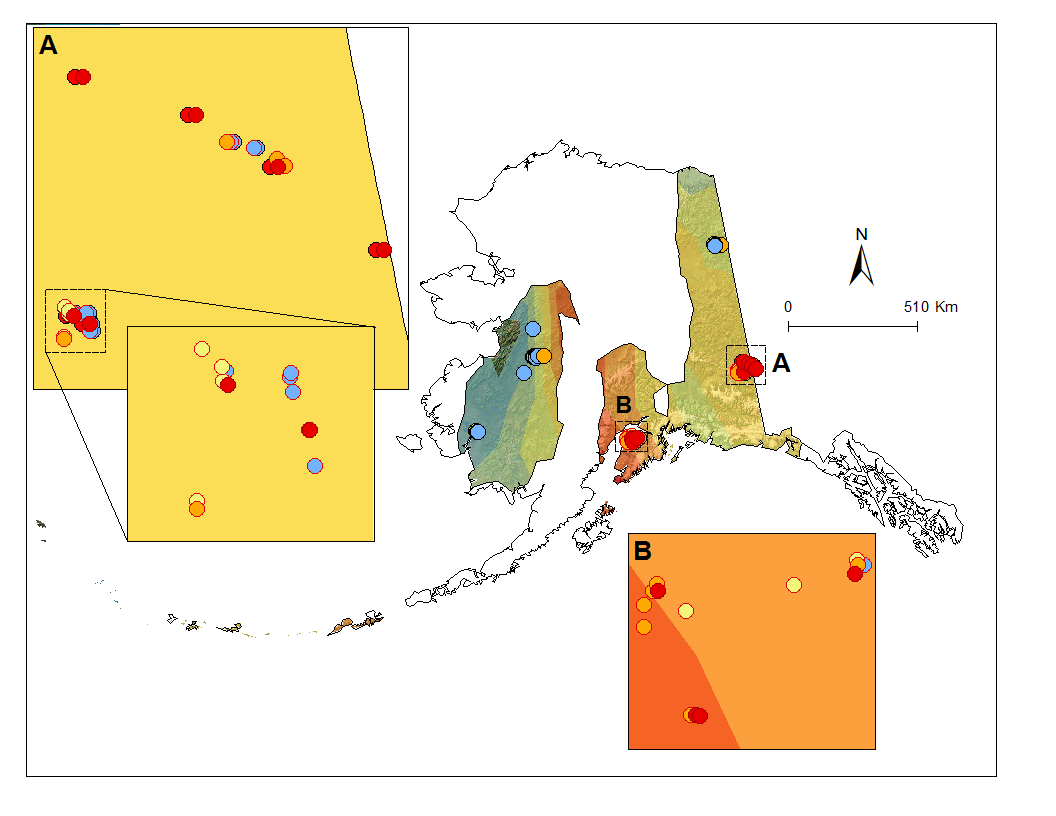

Supplement: Figure S7 — Geographic distribution of amphibian abnormalities with hotspot clusters in Alaska (USFWS Region 7). Shows sites color coded and surface interpolated using the mean abnormality prevalence at each site. Warmer colors represent higher predicted abnormality prevalence (% of frogs abnormal). Sample sites from the 10-year survey are shown as circles; sites in significant hotspot clusters with high abnormality prevalence are indicated by a red circle outline. White polygons mask areas with high standard error (>0.023 prevalence units; Figure S9). (TIF) [file pone.0077467.s007.tif]

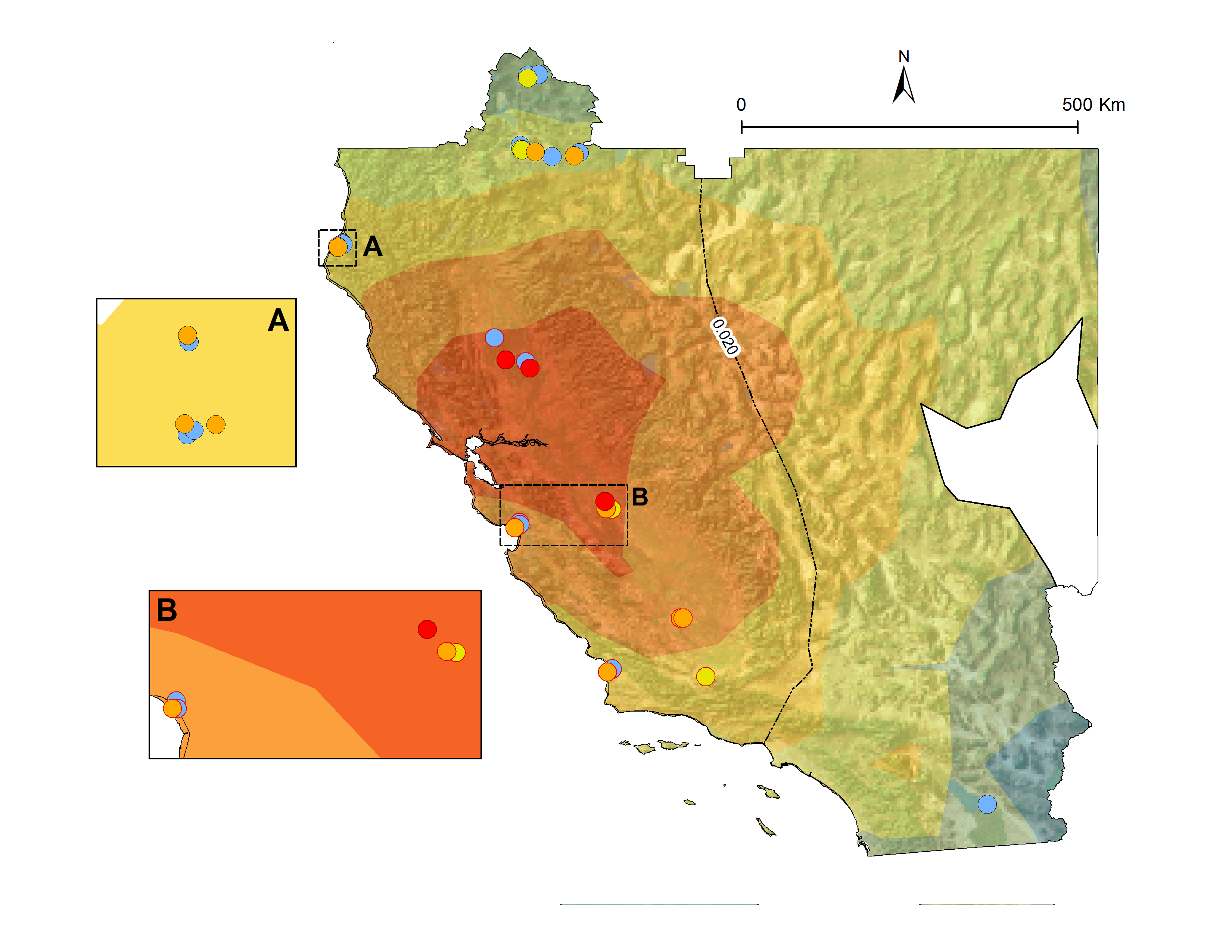

Supplement: Figure S8 — Geographic distribution of amphibian abnormalities with hotspot clusters in California and Nevada (USFWS Region 8). Shows sites color coded and surface interpolated using the mean abnormality prevalence at each site. Warmer colors represent higher predicted abnormality prevalence (% of frogs abnormal). Sample sites from the 10-year survey are shown as circles; sites in significant hotspot clusters with high abnormality prevalence are indicated by a red circle outline. White polygons mask areas with high standard error (>0.023 prevalence units; Figure S9). (TIF) [file pone.0077467.s008.tif]

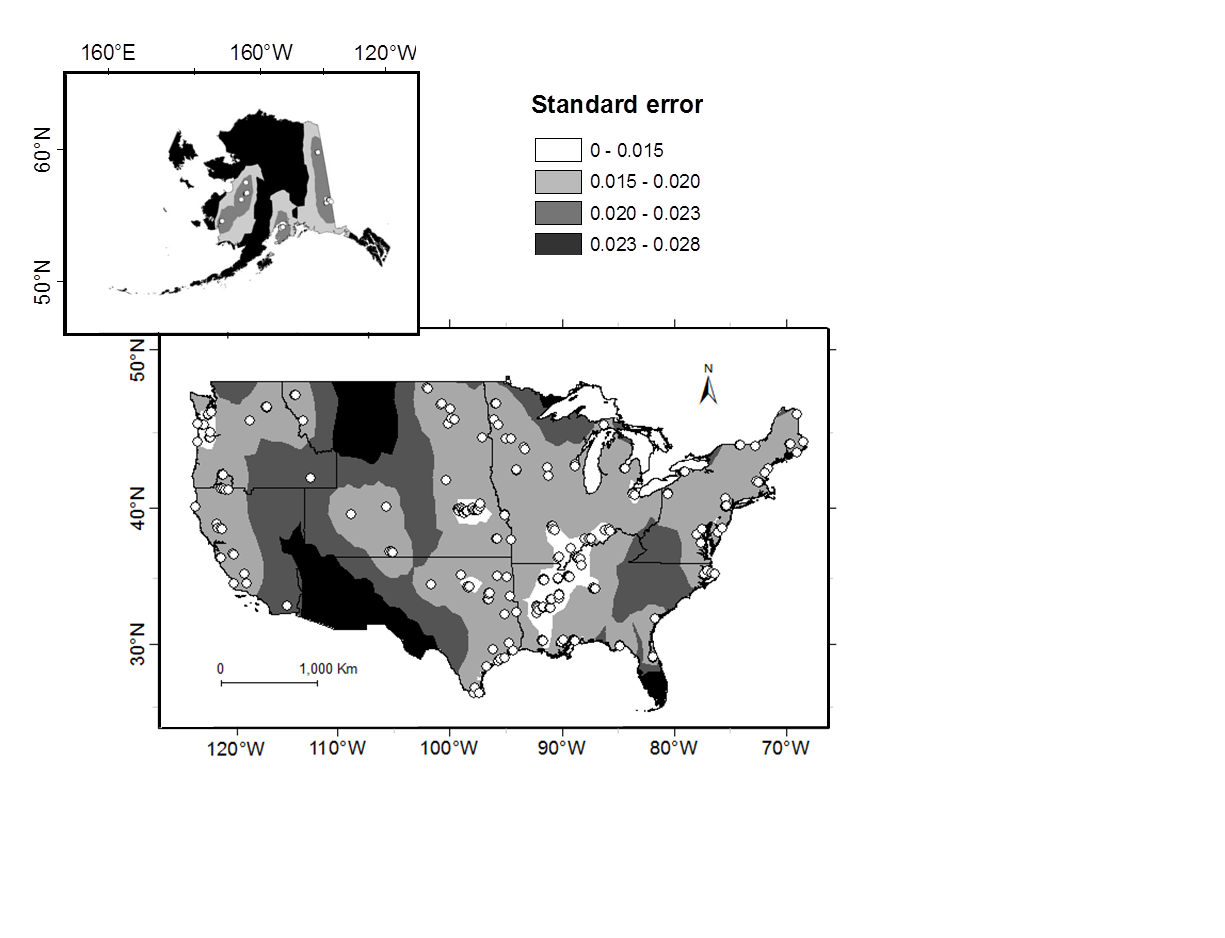

Supplement: Figure S9 — Predicted standard error estimates from the kriging analysis using mean abnormality prevalence at each site. Error is reported in prevalence units (0.01 prevalence unit=1%). (TIF) [file pone.0077467.s009.tif]
