# Supplementary material for: Localized Hotspots Drive Continental Geography of Abnormal Amphibians on U.S. Wildlife Refuges
Source: PLoS One. 2013 Nov 18;8(11):e77467. doi: 10.1371/journal.pone.0077467 (PMC3832516; doi:10.1371/journal.pone.0077467)
Supplement: Figure S10 — Trends in skeletal and eye abnormalities by latitude (left) and longitude (right). We detected significant non-linear trends in skeletal and eye abnormalities along latitude and longitude after accounting for temporal autocorrelation (Table S4). Peaks along trend lines correspond with spatial hotspots from Getis-Ord Gi* analyses that occur in Alaska, the central U.S. (Mississippi River Valley), and California (Figure 2). The solid smooth line is a trend estimated by local regression smoothing (LOESS) using generalized additive modeling and dashed lines represent 95% confidence intervals. The y axes represent the effect of latitude or longitude on skeletal and eye abnormality percentages at sites. (DOCX) [file pone.0077467.s010.docx]

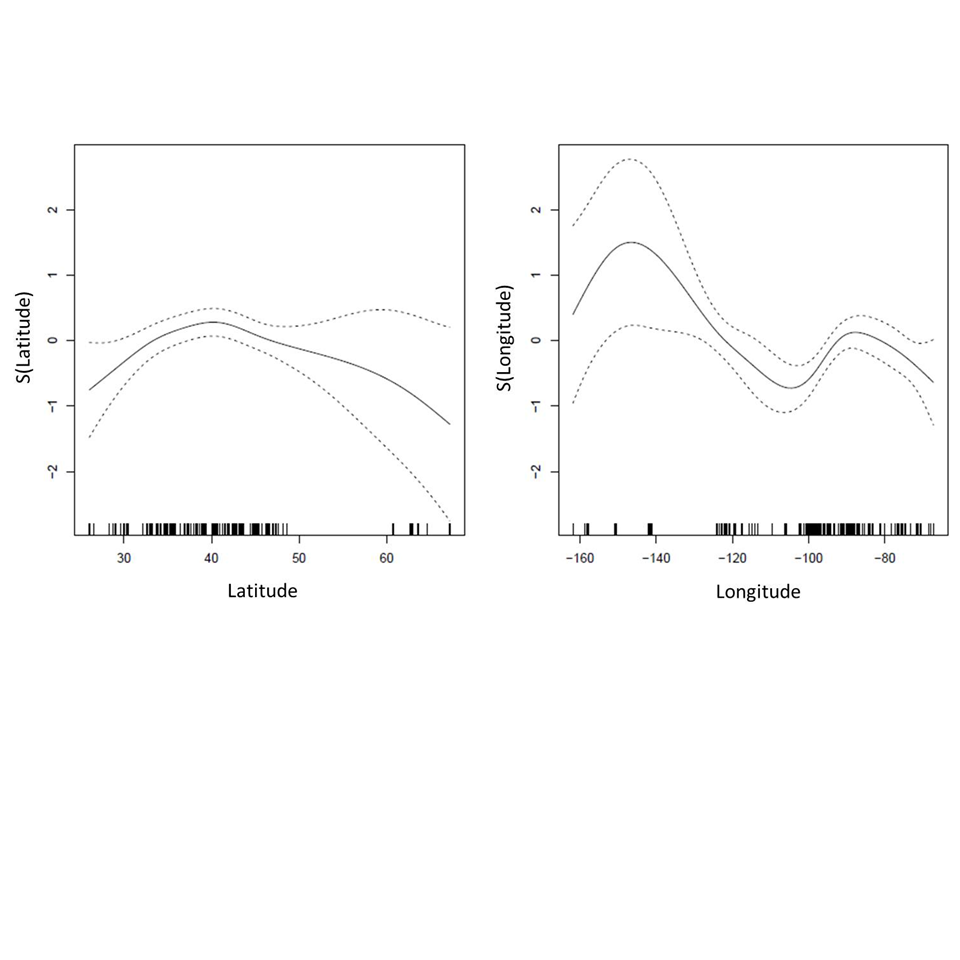
Figure S10.

Trends in skeletal and eye abnormalities by latitude (left) and longitude (right). We detected significant non-linear trends in skeletal and eye abnormalities along latitude and longitude after accounting for temporal autocorrelation (Table S4). Peaks along trend lines correspond with spatial hotspots from Getis-Ord Gi* analyses that occur in Alaska, the central U.S. (Mississippi River Valley), and California (Figure 2). The solid smooth line is a trend estimated by local regression smoothing (LOESS) using generalized additive modeling and dashed lines represent 95% confidence intervals. The y axes represent the effect of latitude or longitude on skeletal and eye abnormality percentages at sites.
