# Supplementary material for: Localized Hotspots Drive Continental Geography of Abnormal Amphibians on U.S. Wildlife Refuges
Source: PLoS One. 2013 Nov 18;8(11):e77467. doi: 10.1371/journal.pone.0077467 (PMC3832516; doi:10.1371/journal.pone.0077467)
Supplement: Figure S11 — Lag matrix model representing distance classes (kilometers) on the x-axes and slope from regressions between inter-site distance and (log) difference in abnormality prevalence for each distance class on the y-axis. Lag-matrix models did not detect a year effect, but significant positive relationships between geographic distance and (log) skeletal and eye abnormalities were detected at approximate inter-region, inter-site, and at one intermediate scale. Significant negative associations were detected at two small spatial scales (0–1 km and 1–5 km). Significant regressions are indicated by filled black symbols. Mean distance between sites within Refuges (Site) was 63 km, between Refuge centroids within regions (Refuge) was 512 km, and between region centroids (Region) was 4,752 km. (DOCX) [file pone.0077467.s011.docx]

Figure S11

Lag matrix model representing distance classes (kilometers) on the x-axes and slope from regressions between inter-site distance and (log) difference in abnormality prevalence for each distance class on the y-axis. Lag-matrix models did not detect a year effect, but significant positive relationships between geographic distance and (log) skeletal and eye abnormalities were detected at approximate inter-region, inter-site, and at one intermediate scale. Significant negative associations were detected at two small spatial scales (0–1 km and 1–5 km). Significant regressions are indicated by filled black symbols. Mean distance between sites within Refuges (Site) was 63 km, between Refuge centroids within regions (Refuge) was 512 km, and between region centroids (Region) was 4,752 km.
