# Supplementary material for: Localized Hotspots Drive Continental Geography of Abnormal Amphibians on U.S. Wildlife Refuges
Source: PLoS One. 2013 Nov 18;8(11):e77467. doi: 10.1371/journal.pone.0077467 (PMC3832516; doi:10.1371/journal.pone.0077467)
Supplement: Figure S12 — USFWS regions. The USFWS has divided the country into eight administrative regions, shown in this figure. These Regions were used to test for coarse-scale spatial differences in abnormality frequency in the hierarchical models. (DOCX) [file pone.0077467.s012.docx]

Figure S12

The USFWS has divided the country into eight administrative regions, shown in this figure. These Regions were used to test for coarse-scale spatial differences in abnormality frequency in the hierarchical models.
