# Supplementary material for: Localized Hotspots Drive Continental Geography of Abnormal Amphibians on U.S. Wildlife Refuges
Source: PLoS One. 2013 Nov 18;8(11):e77467. doi: 10.1371/journal.pone.0077467 (PMC3832516; doi:10.1371/journal.pone.0077467)
Supplement: Table S1 — Hypotheses for amphibian abnormalities (adapted from Johnson et al. 2010). Includes a brief review of each current hypothesis for the causes of skeletal and eye abnormalities in amphibians and a literature cited section. (DOCX) [file pone.0077467.s013.docx]

| Table S1. Hypotheses for amphibian abnormalities (adapted from Johnson et al. 2010). | | |
| --- | --- | --- |
| Hypothesis Name | Summary | Background |
| Null | Abnormalities occur at low frequencies and are caused by random errors in development. | Abnormalities occur in all organisms, but what prevalence is considered “normal” for amphibians? In wild populations, this prevalence is low, generally less than 2% [[1](#_ENREF_1),[2](#_ENREF_2)]. Based on a literature review, others [[3](#_ENREF_3)] proposed a 5% threshold for baseline abnormality levels. Recent studies looking at large numbers of frogs across Canada and the United States support a threshold of 5% or lower: Minnesota (2.5% [[4](#_ENREF_4)], Michigan (0.14% [[5](#_ENREF_5)]), the midwestern and northeastern United States (1.4%-2.6% [[6](#_ENREF_6),[7](#_ENREF_7)]), western Canada (0.2% [[8](#_ENREF_8)]), Vermont (1.6% [[9](#_ENREF_9)]), and Illinois (0.4% [[10](#_ENREF_10)]). It is not clear what fraction of these "baseline" study abnormalities are due to developmental errors versus injuries. |
| Contaminants | Chemicals in the environment cause errors in development that result in a broad spectrum of amphibian malformations. | Amphibians are often cited as indicator organisms, showing heightened sensitivity to environmental conditions before they harm other taxa [[11-13](#_ENREF_11)] . Abnormalities in amphibians could represent a sublethal response to toxic chemicals in their habitat [[14](#_ENREF_14),[15](#_ENREF_15)]. Such contaminant-induced abnormalities have been associated with historic landfills, coal spoils, or other waste disposal areas [[15-21](#_ENREF_15)] or wetlands subjected to localized road or agricultural runoff [[1](#_ENREF_1),[21](#_ENREF_21)]. The teratogenic effects of a number of agricultural chemicals on amphibians have been investigated in controlled experiments [[11](#_ENREF_11),[22-48](#_ENREF_22)]. The effects of these chemicals vary; however, it is clear they can cause skeletal deformities in developing amphibians. A fundamental challenge in investigating the role of contaminants in causing amphibian limb malformations in nature is determining which chemical(s) (or combination thereof) to study. Testing for the full-suite of these compounds and their various breakdown products in wetlands is prohibitively expensive and can be methodologically challenging. |
| Parasites | Parasites infect developing limbs of amphibian larvae causing a variety of abnormalities, commonly multiple limbs, bony triangles, and skin webbings. | This hypothesis was first suggested in 1990 [[49](#_ENREF_49)] when high frequencies of malformed amphibians in a California pond were found to have parasites encysted near the malformed limbs. Subsequent work identified the parasite responsible as *Ribeiroia ondatrae* [[50](#_ENREF_50),[51](#_ENREF_51)], a digenetic trematode with a multi-host life cycle. *Ribeiroia* moves sequentially among freshwater snails, larval amphibians, and finally, birds or, less frequently, mammals [[52](#_ENREF_52)]. Extensive evidence now supports a causal link between *Ribeiroia* infection and limb malformations in amphibians [[53-59](#_ENREF_53)]. Exposure to realistic numbers of *Ribeiroia* cercariae causes increased mortality and severe malformations in frogs, toads, and salamanders. The frequency of malformations induced is often high, and can reach 100% among surviving animals. Malformations and mortality are dose-dependent; higher levels of *Ribeiroia* exposure increase the risk and the severity of malformations produced, as well as the likelihood that the animal dies following exposure. Low levels of infection may or may not cause any obvious pathology. |
| Predators | Predators bite the developing limbs off of amphibian larvae, leading to missing or partially-regenerated, but malformed limbs. | Growing evidence suggests that predators may play an important role in causing certain types of abnormalities in amphibians. Predators like stickleback [[60](#_ENREF_60)], dragonfly larvae [[60](#_ENREF_60),[61](#_ENREF_61)], or leeches [[62](#_ENREF_62),[63](#_ENREF_63)] cause abnormalities by attacking developing tadpole hind limbs without killing the tadpole. In these cases, the abnormalities are dominated by partially and completely missing hind limbs. Predators may create an injury that either results directly in an abnormality (e.g., a missing limb) or creates a malformation during the regeneration process, often lacking obvious scar tissue and with some development of digits at the distal end. |
| UV-B | UV-B radiation causes bilateral and symmetrical shrunken limb abnormalities when larvae are exposed during development. | Declines in the earth’s ozone layer have caused seasonal increases in the level of UV-B penetration, which is suspected to have deleterious effects on aquatic systems [[64-70](#_ENREF_64)] . Because many amphibians deposit their eggs in shallow water, they are perhaps particularly vulnerable to changes in UV-B [[65](#_ENREF_65),[71-73](#_ENREF_71)]. Laboratory and outdoor studies have established that exposure to ambient UV-B can cause high frequencies of limb reductions or deletions in amphibians [[74-76](#_ENREF_74)]. However, the resulting abnormalities were generally bilaterally symmetrical, unlike most field observations, causing the authors to question a direct role of UV-B in explaining recently observed malformations in amphibians [[77](#_ENREF_77)]. Additionally, field studies and risk assessment analyses suggest that the levels of UV-B exposure to which amphibians are exposed in natural wetlands is usually insufficient to induce abnormalities [[78](#_ENREF_78)]. In nature, UV-B is often rapidly attenuated in aquatic ecosystems, owing to dissolved organic carbon in water, and maternal behavior can further buffer offspring from UV-B effects by laying eggs in the shade [[78](#_ENREF_78)]. |
| Multiple Stressors | Multiple stressors (often a combination of biotic and abiotic) like nutrients and parasites, chemicals and predators, or chemicals and UV-B, must co-occur to cause abnormalities, sometimes by complex mechanisms. | Several recent studies have shown abnormalities in nature to have complex chains of causation, that several things must go wrong at once to cause high abnormality frequencies [[14](#_ENREF_14),[79](#_ENREF_79),[80](#_ENREF_80)]. In some cases, chemicals associated with agricultural runoff have increased levels of parasite infection [[79](#_ENREF_79),[80](#_ENREF_80)]. In other cases, relatively constant contamination combines with variable effects due to the timing of other more dynamic stressors like parasites or predators [[14](#_ENREF_14),[81-83](#_ENREF_81)]. In a third example, chemical pollutants may vary in toxicity when they are irradiated with UV-B, which may activate toxic compounds and increase their toxicity, causing increased frequencies of abnormalities [[15](#_ENREF_15),[84-86](#_ENREF_84)]. |
| Species | Either genetics or life historical factors make some species more susceptible to abnormalities than others. | Susceptibilities to limb abnormalities may differ among species and could be related to such genetic or life history characteristics as frog or toad size [[21](#_ENREF_21)], habitat preference [[87](#_ENREF_87)], or susceptibility to parasite infection [[88-90](#_ENREF_88)], or contaminant toxicity [[91-94](#_ENREF_91)], or predator attack [[60](#_ENREF_60),[61](#_ENREF_61),[95](#_ENREF_95)]. |
| Time | Some studies have shown North American abnormality frequencies to be increasing through time. | Historically, severe malformations were uncommon in amphibian populations. Museum studies and resurveys of historic field sites suggest that, in some regions, abnormalities have increased [[10](#_ENREF_10),[96-98](#_ENREF_96)]. Accounts in which >5% of the amphibian population exhibited limb malformations, are extraordinarily rare in the historical literature (1900-1990; [[97](#_ENREF_97)] for review). Collectively, these findings support the hypothesis that recently observed patterns of amphibian malformations deviate from the historical precedent. |
| Climate | Climatic changes like excessive rainfall or drought may cause abnormalities through temperature extremes or increased tadpole densities. | Abnormal amphibian development has been observed when larvae are exposed to extremely high or low temperatures either directly [[99-103](#_ENREF_99)] or through interactions with other stressors [[83](#_ENREF_83),[103-107](#_ENREF_103)]. |
| Cyanobacteria | Harmful bacteria that occupy eutrophic habitats can produce malformation-causing retinoic acids | Retinoic acids are teratogens known to cause malformations in amphibians and other species [[91](#_ENREF_91),[92](#_ENREF_92),[108](#_ENREF_108),[109](#_ENREF_109)]. Recent research has shown that cyanobacteria associated with toxic algal blooms in artificially eutrophic habitats produce retinoic acids which may cause malformations in aquatic species inhabiting these affected areas [[110](#_ENREF_110)]. |

**References**

1. Lannoo MJ (2008) Malformed Frogs: The Collapse of Aquatic Ecosystems: University of California Press. 270 p.

2. Ouellet M (2000) Amphibian Deformities: Current State of Knowledge. In: Ecotoxicology of Amphibians and Reptiles: Society of Environmental Toxicology and Chemistry (SETAC). pp. 617-661.

3. Johnson PTJ, Lunde KB, Ritchie EG, Reaser JK, Launer AE (2001) Morphological abnormality patterns in a California amphibian community. Herpetologica 57: 336-352.

4. Helgen JC, Gernes MC, Kersten SM, Chirhart JW, Canfield JT, et al. (2000) Field investigations of malformed frogs in Minnesota 1993-1997. J Iowa Acad Sci 107: 96-112.

5. Gillilland MG, Muzzall PM (2002) Amphibians, trematodes, and deformities: An overview from southern Michigan. Comp Parasitol 69: 81-85.

6. Converse KA, Mattsson J, Eaton-Poole L (2000) Field surveys of Midwestern and Northeastern Fish and Wildlife Service lands for the presence of abnormal frogs and toads. J Iowa Acad Sci 107: 160-167.

7. Schoff PK, Johnson CM, Schotthoefer AM, Murphy JE, Lieske C, et al. (2003) Prevalence of skeletal and eye malformations in frogs from North-Central United States: Estimations based on collections from randomly selected sites. J Wildlife Dis 39: 510-521.

8. Eaton BR, Eaves S, Stevens C, Puchniak A, Paszkowski CA (2004) Deformity levels in wild populations of the wood frog (*Rana sylvatica*) in three ecoregions of Western Canada. J Herpetol 38: 283-287.

9. Taylor B, Skelly D, Demarchis LK, Slade MD, Galusha D, et al. (2005) Proximity to pollution sources and risk of amphibian limb malformation. Environ Health Persp 113: 1497-1501.

10. Gray RH (2000) Morphological abnormalities in Illinois cricket frogs, *Acris crepitans*, 1968-71. J Iowa Acad Sci 107: 92-95.

11. Cooke AS (1981) Tadpoles as indicators of harmful levels of pollution in the field. Environ Pollut 25: 123-133.

12. Blaustein AR (1994) Chicken Little or Nero's Fiddle - a perspective on declining amphibian populations. Herpetologica 50: 85-97.

13. van der Schalie WH, H.S. G, Bantle JA, De Rosa CT, Finch RA, et al. (1999) Animals as sentinels of human health hazards of environmental chemicals. Environ Health Persp 107: 309-315.

14. Reeves MK, Jensen P, Dolph CL, Holyoak M, Trust KA (2010) Multiple stressors and the cause of amphibian abnormalities. Ecol Monogr 80: 423-440.

15. Bacon JP, Fort CE, Todhunter B, Mathis M, Fort DJ (2013) Effects of multiple chemical, physical, and biological stressors on the incidence and types of abnormalities observed in Bermuda's cane toads (*Rhinella marina*). J Exp Zool B: Mol Dev Evol: DOI: 10.1002/jez.b.22496.

16. Rowe CL, Kinney OM, Congdon JD (1998) Oral deformities in tadpoles of the bullfrog (*Rana catesbeiana)* caused by conditions in a polluted habitat. Copeia 1: 244-246.

17. Hopkins WA, Ray JK, Congdon JD (1998) Incidence and impact of axial malformations in bullfrog larvae (*Rana catesbeiana*) developing in sites impacted by coal combustion byproducts. Am Zool 38: 194A.

18. Hopkins WA, Congdon J, Ray JK (2000) Incidence and impact of axial malformations in larval Bullfrogs (*Rana catesbeiana*) developing in sites polluted by a coal-burning power plant. Environ Toxicol Chem 19: 862-868.

19. Linzey DW, Burroughs J, Hudson L, Marini M, Robertson J, et al. (2003) Role of environmental pollutants on immune functions, parasitic infections, and limb malformations in marine toads and whistling frogs from Bermuda. Int J Environ Heal R 13: 125-148.

20. Fort DJ, Rogers RL, Bacon JP (2006) Deformities in cane toad (*Bufo marinus*) populations in Bermuda: Part II. Progress towards characterization of chemical stressors. Appl Herpetol 3: 143-172.

21. Reeves MK, Dolph CL, Zimmer H, Tjeerdema RS, Trust KA (2008) Road proximity increases risk of skeletal abnormalities in wood frogs from National Wildlife Refuges in Alaska. Environ Health Persp 116: 1009-1014.

22. Berrill M, Bertram S, McGillivray L, Kolohon M, Pauli B (1994) Effects of low concentrations of forest-use pesticides on frog embryos and tadpoles. Environ Toxicol Chem 13: 657-664.

23. Howe GE, Gillis R, Mowbray RC (1998) Effect of chemical synergy and larval stage on the toxicity of atrazine and alachlor to amphibian larvae. Environ Toxicol Chem 17: 519-525.

24. Kloas W, Lutz I, Einspanier R (1999) Amphibians as a model to study endocrine disruptors: II. Estrogenic activity of environmental chemicals in vitro and in vivo. Sci Total Environ 225: 59-68.

25. Maund SJ, Travis KZ, Hendley P, Giddings JM, Solomon KR (2001) Probablistic risk assessment of cotton Pyrethroids: V. combining landscape-level exposures and ecotoxicological effects data to characterize risks. Environ Toxicol Chem 20: 687-692.

26. Bridges CM, Dwyer FJ, D.K. H, WhitesD.W. (2002) Comparative contaminant toxicity: Are amphibian larvae more sensitive than fish? B Environ Contam Tox 69: 562-569.

27. Fort DJ, McLaughlin DW, Rogers RL, Buzzard BO (2002) Effect of endocrine disrupting chemicals on germinal vesicle breakdown in *Xenopus* in vitro. Drug Chem Toxicol 25: 293-308.

28. Osano O, Admiraal W, Otieno D (2002) Developmental disorders in embryos of the frog *Xenopus laevis* induced by chloroacetanilide herbicides and their degradation products. Environ Toxicol Chem 21: 375-379.

29. Sone K, Hinago M, Kitayama A, Morokuma J, Ueno N, et al. (2004) Effects of 17β-estradiol, nonylphenol, and bisphenol-A on developing *Xenopus laevis* embryos. Gen Comp Endocr 138: 228-236.

30. Mendez SIS, Tillitt DE, Rittenhouse TAG, Semlitsch RD (2009) Behavioral response and kinetics of terrestrial atrazine exposure in American Toads (*Bufo americanus*). Arch Environ Con Tox 57: 590-597.

31. Langer-Jaesrich M, Kienle C, Koehler H-R, Gerhardt A (2010) Impairment of trophic interactions between zebrafish (*Danio rerio*) and midge larvae (*Chironomus riparius*) by chlorpyrifos. Ecotoxicology 19: 1294-1301.

32. Schuytema GS, Nebeker AV, Griffis WL, Wilson KN (1991) Teratogenesis, toxicity, and bioconcentration in frogs exposed to Dieldrin. Arch Environ Con Tox 21: 332-350.

33. Schuytema GS, Nebeker AV, Griffis WL (1994) Toxicity of Guthion and Guthion 2S to *Xenopus laevis* embryos. Arch Environ Con Tox 27: 250-255.

34. Schuytema GS, Nebeker AV (1998) Comparative Toxicity of Diuron on Survival and Growth of Pacific Treefrog, Bullfrog, Red-Legged Frog, and African Clawed Frog Embryos and Tadpoles. Arch Env Con Toxicol 34: 370-376.

35. Hall RJ, Mulhern BM (1984) Are anuran amphibians heavy metal accumulators? Vertebrate ecology and systematics: 123-133.

36. Clark K, Hall R (1985) Effects of elevated hydrogen ion and aluminum concentrations on the survival of amphibian embryos and larvae. Can J Zool 63: 116-123.

37. Hall RJ, Henry PFP (1992) Assessing the effects of pesticides on amphibians and reptiles: status and needs. J Herpetol 2: 65-71.

38. Freda J, Taylor DH (1992) Behavioral-response of amphibian larvae to acidic water. J Herpetol 26: 429-433.

39. Taylor SK, Williams ES, Mills KW (1999) Effects of Malathion on disease susceptibility in Woodhouse's toads. J Wildlife Dis 35: 536-541.

40. Christin MS, Gendron AD, Brousseau P, Menard L, Marcogliese DJ, et al. (2003) Effects of agricultural pesticides on the immune system of *Rana pipiens* and on its resistance to parasitic infection. Environ Toxicol Chem 22: 1127-1133.

41. Alvarez R, Honrubia P, Herraez MP (1995) Skeletal malformations induced by the insecticides ZZ-Aphox and Folidol during larval development of *Rana perezi*. Arch Env Cont Tox 28: 349-356.

42. Bridges CM (1999) Predator-prey interactions between two amphibian species: effects of insecticide exposure. Aquatic Ecology 33: 205-211.

43. Bridges CM (2000) Long-term Effects of Pesticide Exposure at Various Life Stages of the Southern Leopard Frog (*Rana sphenocephala*). Arch Env Contam Toxicol 39: 91-96.

44. Bridges CM, Semlitsch RD (2000) Variation in pesticide tolerance of tadpoles among and within species of Ranidae and patterns of amphibian decline. Conserv Biol 14: 1490-1499.

45. Bridges CM, Semlitsch RD (2001) Genetic variation in insecticide tolerance in a population of southern leopard frogs (*Rana sphenocephala*): Implications for amphibian conservation. Copeia: 7-13.

46. Sparling DW, Fellers GM, McConnell LL (2001) Pesticides and amphibian population declines in California, USA. Environ Toxicol Chem 20: 1591-1595.

47. Saura-Mas S, Boone MD, Bridges CM (2002) Evaluation of direct effects of an insecticide on gray treefrogs: laboratory and field trials. J Herpetol 36: 715-719.

48. Boone MD, Bridges CM (2003) Effects of Carbaryl on Green Frog (*Rana clamitans*) tadpoles: Timing of exposure versus multiple exposures. Environ Toxicol Chem 22: 2695-2702.

49. Sessions SK, Ruth SB (1990) Explanation for naturally occurring supernumerary limbs in amphibians. J Exp Zool 254: 38-47.

50. Johnson PTJ, Lunde KB, Ritchie EG, Launer AE (1999) The effect of trematode infection on amphibian limb development and survivorship. Science 284: 802-804.

51. Johnson PTJ, Sutherland DR (2003) Amphibian deformities and *Ribeiroia* infection: an emerging helminthiasis. Trends Parasitol 19: 332-335.

52. Johnson PT, Sutherland DR, Kinsella JM, Lunde KB (2004) Review of the trematode genus *Ribieroia* (Psilostomidae): Ecology, life history, and pathogenesis with special emphasis on the amphibian malformation problem. Adv Parasit 57: 191-253.

53. Johnson PT, Lunde KB, Haight RW, Bowerman J, Blaustein AR (2001) *Ribeiroia ondatrae* (Trematoda: Digenea) infection induces severe limb malformations in western toads (*Bufo boreas*). Can J Zool 79: 370-379.

54. Johnson PTJ, Preu ER, Sutherland DR, Romansic JM, Han B, et al. (2006) Adding infection to injury: Synergistic effects of predation and parasitism on amphibian malformations. Ecology 87: 2227-2235.

55. Johnson PTJ (2006) Amphibian diversity: Decimation by disease. Proc Nat Acad Sci USA 103: 3011-3012.

56. Johnson PTJ, Hartson RB, Larson DJ, Sutherland DR (2008) Diversity and disease: community structure drives parasite transmission and host fitness. Ecol Lett 11: 1017-1026.

57. Schotthoefer AM, Koehler AV, Meteyer CU, Cole RA (2003) Influence of *Ribeiroia ondatrae* (Trematoda : Digenea) infection on limb development and survival of northern leopard frogs (*Rana pipiens*): effects of host stage and parasite-exposure level. Can J Zool 81: 1144-1153.

58. Stopper GF, Hecker L, Franssen A, Sessions SK (2002) How trematodes cause limb deformities in amphibians. J Exp Zool 294: 252-263.

59. Kiesecker JM (2002) Synergism between trematode infection and pesticide exposure: A link to amphibian limb deformities in nature? Proc Nat Acad Sci USA 99: 9900-9904.

60. Bowerman J, Johnson PTJ, Bowerman T (2010) Sublethal predators and their injured prey: linking aquatic predators and severe limb abnormalities in amphibians. Ecology 91: 242-251.

61. Ballengée B, Sessions SK (2009) Explanation for missing limbs in deformed amphibians. J Exp Zool B: Mol Dev Evol 312B: 770-779.

62. Bohl E (1997) Limb deformities of amphibian larvae in Aufsess (Upper Franconia): attempt to determine causes. Münich Contr Wast Fish River Bio 50: 160-189.

63. Viertel B, Veith M. Predation by leeches and regeneration, a factor in larval development of *Bufo bufo* (L.); 1992 1991; Budapest. pp. 479-484.

64. Licht LE, Grant KP (1997) The effects of ultraviolet radiation on the biology of amphibians. Am Zool 37: 137-145.

65. Blaustein AR, Kiesecker JM, Chivers DP, Hokit DG, Marco A, et al. (1998) Effects of ultraviolet radiation on amphibians: Field experiments. Am Zool 38: 799-812.

66. Hader DP, Kumar HD, Smith RC, Worrest RC (1998) Effects on aquatic ecosystems. J Photoch Photobio B 46: 53-68.

67. Blaustein AR, Johnson PTJ (2003) The complexity of deformed amphibians. Front Ecol Environ 1: 87-94.

68. Collins JP, Storfer A (2003) Global amphibian declines: sorting the hypotheses. Diversity Distrib 9: 89-98.

69. Blaustein AR, Romansic JM, Kiesecker JM, Hatch AC (2003) Ultraviolet radiation, toxic chemicals and amphibian population declines. Diversity Distrib 9: 123-140.

70. Sinha RP, Hader DP (2002) Life under solar UV radiation in aquatic organisms. Space Life Sciences: In: Extraterrestrial Organic Chemistry, UV Radiation on Biological Evolution, and Planetary Protection. pp. 1547-1556.

71. Blaustein AR, Hoffman PD, Hokit DG, Kiesecker JM, Walls SC, et al. (1994) UV repair and resistance to solar UV-B in amphibian eggs: A link to population declines? Proc Nat Acad Sci USA 91: 1791-1795.

72. Blaustein AR, Kiesecker JM, Chivers DP, Anthony RG (1997) Ambient UV-B radiation causes deformities in amphibian embryos. Proc Nat Acad Sci USA 94: 13735-13737.

73. Crump D, Berrrill M, Coulson D, Lean D, McGillivray L, et al. (1999) Sensitivity of amphibian embryos, tadpoles, and larvae to enhanced UV-B radiation in natural pond conditions. Can J Zool 77: 1956-1966.

74. Ankley GT, Diamond SE, Tietge JE, Holcombe GW, Jensen KM, et al. (2002) Assessment of the risk of solar ultraviolet radiation to amphibians. I. dose-dependent induction of hindlimb malformations in the northern leopard frog (*Rana pipiens*). Environ Sci Technol 36: 2853-2858.

75. Diamond SA, Peterson GS, Tietge JE, Ankley GT (2002) Assessment of the risk of solar ultraviolet radiation to amphibians. III. Prediction of impacts in selected northern midwestern wetlands. Environ Sci Technol 36: 2866-2874.

76. Peterson GS, Johnson LB, Axler RP, Diamond SA (2002) Assessment of the risk of solar ultraviolet radiation to amphibians. II. In situ characterization of exposure in amphibian habitats. Environ Sci Technol 36: 2859-2865.

77. Ankley GA, Degitz SJ, Diamond SA, Tietge JE (2004) Assessment of environmental stressors potentially responsible for malformations in North American anuran amphibians. Ecotox Environ Safe 58: 7-16.

78. Palen WJ, Schindler DE (2010) Water clarity, maternal behavior, and physiology combine to eliminate UV radiation risk to amphibians in a montane landscape. Proc Nat Acad Sci USA 107: 9701-9706.

79. Rohr JR, Schotthoefer AM, Raffel TR, Carrick HJ, Halstead N, et al. (2008) Agrochemicals increase trematode infections in a declining amphibian species. Nature 455: 1235-1239.

80. Johnson PTJ, Chase JM, Dosch KL, Hartson RB, Gross JA, et al. (2007) Aquatic eutrophication promotes pathogenic infection in amphibians. Proc Nat Acad Sci USA 104: 15781-15786.

81. Reeves MK, Perdue M, Blakemore GD, Rinella DJ, Holyoak M (2011) Twice as easy to catch? A toxicant and a predator cue cause additive reductions in larval amphibian activity. Ecosphere 2: art72.

82. Johnson PTJ, McKenzie VJ, Peterson AC, Kerby JL, Brown J, et al. (2011) Regional decline of an iconic amphibian associated with elevation, land-use change, and invasive species. Conserv Biol 25: 556-566.

83. Paull SH, Johnson PTJ (2011) High temperature enhances host pathology in a snail-trematode system: possible consequences of climate change for the emergence of disease. Freshwater Biol 56: 767-778.

84. Hatch AC, Burton GAJ (1998) Effects of photoinduced toxicity of fluoranthene on amphibian embryos and larvae. Environ Toxicol Chem 17: 1777-1785.

85. LaClair JJ, Bantle JA, Dumont J (1998) Photoproducts and metabolites of a common insect growth regulator produce developmental deformities in *Xenopus*. Environ Sci Technol 32: 1453-1461.

86. Bilski P, Burkhart JG, Chignell CF (2003) Photochemical characterization of water samples from Minnesota and Vermont sites with malformed frogs: potential influence of photosensitization by singlet molecular oxygen and free radicals on aquatic toxicity. Aquat Toxicol 65: 229-241.

87. Hoppe DM (2005) Malformed frogs in Minnesota: History and interspecific differences. Amphibian Declines: The Conservation Status of United States Species. Berkeley: University of California Press. pp. 103-108.

88. Johnson PTJ, Rohr JR, Hoverman JT, Kellermanns E, Bowerman J, et al. (2012) Living fast and dying of infection: host life history drives interspecific variation in infection and disease risk. Ecol Lett 15: 235-242.

89. Johnson PTJ, Preston DL, Hoverman JT, Henderson JS, Paull SH, et al. (2012) Species diversity reduces parasite infection through cross-generational effects on host abundance. Ecology 93: 56-64.

90. Johnson PTJ, Hartson RB (2009) All hosts are not equal: explaining differential patterns of malformations in an amphibian community. J Anim Ecol 78: 191-201.

91. Gardiner DM, Hoppe DM (1999) Environmentally induced limb malformations in mink frogs (*Rana septentrionalis*). J Exp Zool 284: 207-216.

92. Degitz SJ, Kosian PA, Makynen EA, Jensen KM, Ankley GT (2000) Stage- and species-specific developmental toxicity of all-trans retinoic acid in four native North American ranids and *Xenopus laevis*. Toxicol Sci 57: 264-274.

93. Degitz SJ, Holcombe GW, Kosian PA, Tietge JE, Durhan EJ, et al. (2003) Comparing the effects of stage and duration of retinoic acid exposure on amphibian limb development: Chronic exposure results in mortality, not limb malformations. Toxicol Sci 74: 139-146.

94. Gardiner D, Ndayibagira A, Grün F, Blumberg B (2003) Deformed frogs and environmental retinoids. Pure and Applied Chemistry 75: 2263-2273.

95. Johnson PTJ, Bowerman J (2010) Do predators cause frog deformities? The need for an eco-epidemiological approach. J Exp Zool B: Mol Dev Evol 314B: 515-518.

96. Hoppe DM (2000) History of Minnesota frog abnormalities: do recent findings represent a new phenomenon? J Iowa Acad Sci 107: 86-89.

97. Johnson PTJ, Lunde KB, Zelmer DA, Werner JK (2003) Limb deformities as an emerging parasitic disease in amphibians: Evidence from museum specimens and resurvey data. Conserv Biol 17: 1724-1737.

98. McCallum ML, Trauth SE (2003) Museum study of northern cricket frog (*Acris crepitans*) abnormalities in Arkansas: Upward trends and distributions. J Wildlife Dis 39: 510-521.

99. Witschi E (1929) Studies in sex differentiation and sex determination in amphibians: II. Sex reversal in female tadpoles of *Rana sylvatica* following the application of high temperature. J Exp Zool 52: 267-291.

100. Cupp PVJ (1980) Thermal tolerance of five salientian amphibians during development and metamorphosis. Herpetologica 36: 234-244.

101. Hayes TB (1998) Sex determination and primary sex differentiation in amphibians: genetic and developmental mechanisms. J Exp Zool 281: 373-399.

102. Garcia TS, Straus R, Sih A (2003) Temperature and ontogenetic effects on color change in the larval salamander species *Ambystoma barbouri* and *Ambystoma texanum*. Can J Zool 81: 710-715.

103. Kaplan RH, Phillips PC (2006) Ecological and developmental context of natural selection: Maternal effects and thermally induced plasticity in the frog *Bombina orientalis*. Evolution 60: 142-156.

104. Moore MK, Townsend VR (1998) The interaction of temperature, dissolved oxygen and predation pressure in an aquatic predator-prey system. Oikos 81: 329-336.

105. Boone MD, Bridges CM (1999) The effect of temperature on the potency of Carbaryl for survival of tadpoles of the green frog (*Rana clamitans*). Environ Toxicol Chem 18: 1482-1484.

106. Anderson MT, Kiesecker JM, Chivers DP, Blaustein AR (2001) The direct and indirect effects of temperature on a predator-prey relationship. Can J Zool 79: 1834-1841.

107. Paull SH, LaFonte BE, Johnson PTJ (2012) Temperature-driven shifts in a host-parasite interaction drive nonlinear changes in disease risk. Global Change Biology 18: 3558-3567.

108. Bryant SV, Gardiner DM (1992) Retinoic acid, local cell-cell interactions and pattern formation in vertebrate limbs. Dev Biol 152: 1-25.

109. Carey C, Bryant C (1995) Possible interrelations among environmental toxicants, amphibian development, and decline of amphibian populations. Environ Health Persp 103: 13-17.

110. Wu X, Jiang J, Wan Y, Giesy JP, Hu J (2012) Cyanobacteria blooms produce teratogenic retinoic acids. Proc Nat Acad Sci USA: DOI 10.1073/pnas.1200062109.
