# Supplementary material for: Localized Hotspots Drive Continental Geography of Abnormal Amphibians on U.S. Wildlife Refuges
Source: PLoS One. 2013 Nov 18;8(11):e77467. doi: 10.1371/journal.pone.0077467 (PMC3832516; doi:10.1371/journal.pone.0077467)
Supplement: Table S2 — Percentile ranks of skeletal and eye abnormalities in collection events in the coredataset (n=675 Collections and 48,081 amphibians). (DOCX) [file pone.0077467.s014.docx]

Table S2

Percentile ranks of skeletal and eye abnormalities in collection events in the *core dataset* (n=675 Collections and 48,081 amphibians).

| Percentile Rank | Skeletal and Eye Abnormalities (%) |
| --- | --- |
| 0 | 0.0 |
| 5 | 0.0 |
| 10 | 0.0 |
| 15 | 0.0 |
| 20 | 0.0 |
| 25 | 0.0 |
| 30 | 0.0 |
| 35 | 1.0 |
| 40 | 1.3 |
| 45 | 1.8 |
| 50 | 1.9 |
| 55 | 2.0 |
| 60 | 2.6 |
| 65 | 3.3 |
| 70 | 3.9 |
| 75 | 4.7 |
| 80 | 5.6 |
| 85 | 6.3 |
| 90 | 8.3 |
| 95 | 11.5 |
| 100 | 40.0 |
