# Supplementary material for: Localized Hotspots Drive Continental Geography of Abnormal Amphibians on U.S. Wildlife Refuges
Source: PLoS One. 2013 Nov 18;8(11):e77467. doi: 10.1371/journal.pone.0077467 (PMC3832516; doi:10.1371/journal.pone.0077467)
Supplement: Table S3 — Abnormalities found during field surveys. Data presented are for the core dataset. Some individuals had more than one distinct abnormality. Bolded rows are summary data. (DOCX) [file pone.0077467.s015.docx]

Table S3

Abnormalities found during field surveys. Data presented are for the *core dataset*. Some individuals had more than one distinct abnormality. Bolded rows are summary data.

| **Abnormality** | **Total Number of Abnormalities** | **Percentage** |
| --- | --- | --- |
| **EYE ABNORMALITIES** | **190** | **10.2** |
| Anophthalmia (missing eye) | 51 | 2.7 |
| Microphthalmia (small eye) | 48 | 2.6 |
| Unpigmented iris (black eye) | 50 | 2.7 |
| Other | 41 | 2.2 |
| **SKELETAL ABNORMALITIES (INJURIES)** | **217** | **11.6** |
| Amelia Fully Removed/Bloody | 4 | 0.2 |
| Brachydactyly Bone Protruding or Blood at Stump | 68 | 3.6 |
| Digits Curled Smashed with Blood | 9 | 0.5 |
| Ectrodactyly Bone Protruding or Blood at Stump | 18 | 1.0 |
| Ectromelia Bone Protruding Blood at Stump | 81 | 4.3 |
| Skeletal Injury other | 37 | 2.0 |
| **SKELETAL ABNORMALITIES (UNCLEAR ORIGIN)** | **1461** | **78.2** |
| Amelia (completely missing limb) | 65 | 3.5 |
| Anteversion (twisted long bones) | 10 | 0.5 |
| Appendage Dislocated broken dangle | 56 | 3.0 |
| Brachydactyly (short digits) | 319 | 17.1 |
| Brachygnathia (short jaw) | 52 | 2.8 |
| Clinodactyly (bent digits) | 2 | 0.1 |
| Cutaneous fusion (skin webbing) | 6 | 0.3 |
| Ectrodactyly (missing digits) | 156 | 8.4 |
| Ectromelia (partially missing limb) | 378 | 20.2 |
| Hemimelia | 65 | 3.5 |
| Kinked tail | 15 | 0.8 |
| Microcephaly (shrunken head or blunt snout) | 2 | 0.1 |
| Micromelia (shrunken limb or limb element) | 61 | 3.3 |
| Non Flexible limb | 3 | 0.2 |
| One Limb thinner | 7 | 0.4 |
| Polydactyly (extra digits) | 15 | 0.8 |
| Polymelia (extra limb) | 12 | 0.6 |
| Polyphalangy | 5 | 0.3 |
| Scoliosis (curved spine) | 6 | 0.3 |
| Syndactyly (digits fused) | 74 | 4.0 |
| Taumelia (bone bridge or triangle) | 15 | 0.8 |
| Skeletal abnormality other | 137 | 7.3 |
| **TOTAL ABNORMALITIES** | **1868** |  |
| **TOTAL INDIVIDUALS EXAMINED** | **48,081** |  |
