# Supplementary material for: Localized Hotspots Drive Continental Geography of Abnormal Amphibians on U.S. Wildlife Refuges
Source: PLoS One. 2013 Nov 18;8(11):e77467. doi: 10.1371/journal.pone.0077467 (PMC3832516; doi:10.1371/journal.pone.0077467)
Supplement: Table S4 — Results from generalized additive mixed modeling of skeletal and eye abnormalities. Edf: equivalent degrees of freedom. Edf values closer to 1 suggest a linear relationship, and larger edf values correspond to increasingly nonlinear relationships. (DOCX) [file pone.0077467.s016.docx]

Table S4

Results from generalized additive mixed modeling of skeletal and eye abnormalities. Edf: equivalent degrees of freedom. Edf values closer to 1 suggest a linear relationship, and larger edf values correspond to increasingly nonlinear relationships.

|  | | | |
| --- | --- | --- | --- |
|  | edf | F | p |
| s(latitude) | 3.44 | 3.85 | 0.007 |
| s(longitude) | 6.09 | 4.96 | <0.001 |
