# Supplementary material for: Localized Hotspots Drive Continental Geography of Abnormal Amphibians on U.S. Wildlife Refuges
Source: PLoS One. 2013 Nov 18;8(11):e77467. doi: 10.1371/journal.pone.0077467 (PMC3832516; doi:10.1371/journal.pone.0077467)
Supplement: Table S5 — Numbers of individuals of each amphibian species sampled in the coredataset. On a national basis, we identified 32 amphibians to species. An additional 5 groupings summarize amphibians only to genus. (DOCX) [file pone.0077467.s017.docx]

Table S5

Ranking of the numbers of individuals of amphibian species sampled in the *core dataset* 2000–2009. On a national basis, we identified 32 amphibians to species. An additional 5 groupings summarize amphibians identified only to genus.

| **Scientific Name^1^** | **Common Name^1^** | **Total** |
| --- | --- | --- |
| *Lithobates pipiens^2^* | Northern Leopard Frog | 10,414 |
| *Lithobates sphenocephalus^2, 3^* | Southern Leopard Frog | 8,247 |
| *Lithobates sylvaticus^2,4^* | Wood Frog | 4,623 |
| *Lithobates blairi^2^* | Plains Leopard Frog | 3,552 |
| *Lithobates clamitans^2^* | Green Frog | 2,922 |
| *Rana aurora* | Northern Red-Legged Frog | 2,685 |
| *Lithobates catesbeiana^2^* | Bullfrog | 2,163 |
| *Anaxyrus fowleri^5^* | Fowler’s Toad | 2,008 |
| *Anaxyrus sp.^5^* | Unknown Toad Species | 1,912 |
| *Acris crepitans* | Northern Cricket Frog | 1,222 |
| *Pseudacris triseriata* | Western Chorus Frog | 963 |
| *Anaxyrus boreas^5^* | Boreal Toad | 925 |
| *Pseudacris regilla* | Pacific Chorus Frog | 865 |
| *Anaxyrus woodhousii^5^* | Woodhouse’s Toad | 684 |
| *Rana luteiventris* | Columbia Spotted Frog | 490 |
| *Pseudacris feriarum* | Upland Chorus Frog | 470 |
| *Pseudacris maculata* | Boreal Chorus Frog | 426 |
| *Hyla chrysocelis or versicolor* | Cope’s Gray Treefrog/Gray Treefrog | 384 |
| *Acris gryllus* | Southern Cricket Frog | 364 |
| *Lithobates berlandieri^2^* | Rio Grande Leopard Frog | 293 |
| *Hyla cinerea* | Green Treefrog | 293 |
| *Rana pretiosa* | Oregon Spotted Frog | 284 |
| *Lithobates septentrionalis^2^* | Mink Frog | 273 |
| *Pseudacris crucifer* | Northern Spring Peeper | 250 |
| *Acris* sp. | Unknown Cricket Frog Species | 256 |
| *Hyla* sp. | Unknown Treefrog Species | 243 |
| *Hyla femoralis* | Pine Woods Tree Frog | 155 |
| *Acris blanchardi* | Blanchard’s Cricket Frog | 115 |
| *Rana draytonii* | California Red Legged Frog | 99 |
| *Bufo quercicus* | Oak Toad | 76 |
| *Pseudacris ocularis* | Little Grass Frog | 75 |
| *Rana palustris* | Pickerel Frog | 75 |
| *Gastrophryne carolinensis* | Eastern Narrowmouth Toad | 69 |
| *Rana circulosa* | Northern Crawfish Frog | 55 |
| *Bufo americanus* | American Toad | 51 |
| *Pseudacris sp.* | Unknown Chorus Frog Species | 50 |
| *Rana sp.* | Unknown True Frog Species | 50 |
| ^1^ Source: Crother (2012).  ^2^ Genus name changed in several *Rana* species to *Lithobates* in 2008.  ^3^ Species name changed from *sphenocephala* to *sphenocephalus* in 2008.  ^4^ Species name changed from *sylvatica* to *sylvaticus* in 2008.  ^5^ Genus name changed in several *Bufo* species to *Anaxyrus* in 2008. | |  |
