# Supplementary material for: Localized Hotspots Drive Continental Geography of Abnormal Amphibians on U.S. Wildlife Refuges
Source: PLoS One. 2013 Nov 18;8(11):e77467. doi: 10.1371/journal.pone.0077467 (PMC3832516; doi:10.1371/journal.pone.0077467)
Supplement: Table S6 — Summary of random effects model structures evaluated. (DOCX) [file pone.0077467.s018.docx]

Table S6

Summary of random effects model structures evaluated.

| **Model** | **Df** | **AIC** | **Δ AIC** |
| --- | --- | --- | --- |
| sk_eye ~ 1 + (1 \| site.year.species) | 2 | 1422 | 0 |
| sk_eye ~ 1 + (1 \| site.year) | 2 | 1429 | -6 |
| sk_eye ~ 1 + (1 \| yrfactor) + (1 \| regionfactor/refuge/site_id/species) | 6 | 1447 | -25 |
| sk_eye ~ 1 + (1 \| yrfactor) + (1 \| regionfactor/site_id/species) | 5 | 1470 | -48 |
| sk_eye ~ 1 + (yrfactor \| site.species) | 56 | 1490 | -68 |
| sk_eye ~ 1 + (yrfactor \| site_id) | 56 | 1491 | -69 |
| sk_eye ~ 1 + (1 \| site.species) | 2 | 1500 | -77 |
| sk_eye ~ 1 + (1 \| yrfactor) + (1 \| regionfactor) + (1 \| site_id) + (1 \| species) | 5 | 1510 | -88 |
| sk_eye ~ 1 + (yrfactor \| site.year) | 56 | 1525 | -103 |
| sk_eye ~ 1 + (1 \| yrfactor) + (1 \| site_id) | 3 | 1536 | -114 |
| sk_eye ~ 1 + (1 \| yrfactor) + (1 \| site_id) + (1 \| species) | 4 | 1537 | -115 |
| sk_eye ~ 1 + (yrfactor \| site_id/species) | 111 | 1584 | -161 |
| sk_eye ~ 1 + (1 \| yrfactor) + (1 \| refuge) | 3 | 1750 | -327 |
