# Supplementary material for: Localized Hotspots Drive Continental Geography of Abnormal Amphibians on U.S. Wildlife Refuges
Source: PLoS One. 2013 Nov 18;8(11):e77467. doi: 10.1371/journal.pone.0077467 (PMC3832516; doi:10.1371/journal.pone.0077467)
Supplement: Text S1 — Supplemental Methods and Discussion. This file includes additional methodological descriptions for abnormality classification, data management, spatially explicit analyses, spatially implicit analyses, power law analyses, and additional discussion of the previously identified Minnesota and Vermont hotspots and how they relate to this dataset. (DOCX) [file pone.0077467.s019.docx]

**Text S1. Supplemental Text**

**Methods**

**Abnormality classification**

Abnormality is a general term referring to any gross deviation from the normal range in morphological variation. To be consistent with other surveys, we focused on the prevalence of skeletal and/or eye abnormalities and excluded surficial abnormalities (i.e., not affecting the skeleton) that appeared to result from traumatic injuries (wounds) or were viewed as signs of infection [[1](#_ENREF_1)]. The term abnormality includes both malformations (permanent structural defects caused by abnormal development), and deformities (alterations, such as amputation, to an otherwise correctly formed organ or structure). We documented all types of skeletal and eye abnormalities because both malformations and deformities can be detrimental to individual anurans and diagnosis is difficult, especially in the field (Table S3). We emphasized the combined prevalence of skeletal and eye abnormalities in our analyses and summarized them as a single category to ensure our results were comparable with most published articles on this topic.

**Database**

All field data were entered into the Abnormal Amphibian Online Database, which underwent rigorous quality control by the USFWS national database coordinator and each USFWS regional coordinator. The database was designed to perform queries and facilitate data management at the national and regional level, and contains information for all amphibians examined by the Abnormal Amphibian Monitoring Program (AAMP).

**Spatially explicit analyses**

***Identification of significant hotspot clusters*:** We used the following formula to calculate the Getis-Ord Gi* statistic:

,

where *x_j_* is the attribute value for feature *j*, *w_i,j_* is the spatial weight between *i* and *j*, and *n* is equal to the number of features. ***Lag matrix models to assess the scale of spatial autocorrelation:*** We used multiple regression on distance matrices [[2](#_ENREF_2),[3](#_ENREF_3)] to evaluate the distance at which abnormalities clustered. We created a distance matrix for each of nine distance classes; three of these approximated the average distance between sites within Refuges, between Refuge centroids within regions, and between region centroids to test whether abnormalities clustered at the site scale, Refuge scale, or regional scale. Distance matrices were collapsed into vectors [[3](#_ENREF_3)], and regressed against the (natural) log-transformed difference in abnormality frequency between sites. Significance at each distance class was determined by permutation, where predictors were randomized and response variables were held constant for 10,000 iterations to create a null distribution. Regression parameters were significant if they exceeded two standard deviations of the mean of the null distribution.

**Spatially implicit analyses**

***Locational factors:*** To test for evidence of locational effects on abnormality frequency, we used three categorical predictors that tested space at a small, medium, and large scale. The smallest scale was the Site, the individual wetland at which collection events occurred. The factor Refuge coded collection events by the USFWS Refuge in which the Site was located. Region assigned the collection to the USFWS administrative region in which it was located (Figure S4). ***Time factor:*** The time factor in this analysis was the calendar year in which collections were made. It tested for the presence of synchronous interannual differences in abnormality frequency at continental or regional scales. To test for significant differences in the temporal occurrence of abnormalities, we assigned to each collection event the sampling year in which it occurred. We treated year as a categorical variable, estimating a mean value for abnormality frequency in each year. ***Species factor:*** The species factor tested whether some species were more frequently abnormal than others. Our data set included information about abnormalities in at least 32 species, but some species had high sample numbers whereas others were less common (Table S5). We did not have confidence in estimates made for species where we had examined fewer than 500 animals in the field, so we combined species with fewer than 500 animals into groups by genus (e.g. *Anaxyrus* other). We then tested species (and genera for <500 animals per species) as a categorical predictor of abnormality frequency. ***Variance components analysis:*** Finally, we performed a simple variance components analysis, which enabled us to apportion variation in skeletal and eye abnormality prevalence to each of these individual factors (Figure 3). Variance estimates were made using a GLMM model (lme4 package in R [[4](#_ENREF_4)]) and specifying each factor as an individual random effect in an intercept only model (i.e., skeletal+eye = 1+ (1|yrfactor) + (1|species) + (1|regionfactor) + (1|Refuge) + (1|site)) using a binomial distribution of sampling error, weighting estimates for sample number, and using restricted maximum likelihood for variance estimation. ***Effect of spatial autocorrelation on spatially implicit models:*** Because we detected clustering of high-abnormality sites, we evaluated whether spatial structure should be included in spatially implicit models to avoid potential problems that can arise with underlying spatial autocorrelation [[5](#_ENREF_5),[6](#_ENREF_6)]. Using the MASS library in R, we evaluated whether adding a spatially structured error term significantly improved model fit. We used simulation to compare between two GLMMs with region as a fixed factor and the same random effect structure used in spatially implicit models (site.year) using penalized quasi-likelihood to estimate parameters. The spatial model also incorporated an exponential correlation structure as a function of latitude and longitude. A significant improvement in fit by adding a correlation structure was assessed by comparing a distribution of difference in model fit between 500 spatial and non-spatial models. We considered a spatial model necessary when the improvement in model fit by using a spatially correlated error structure was significantly better than random model improvement from simulations. Adding a spatially structured error term did not improve model fit for spatially implicit models, so we report models without spatially correlated error.

**Power Law Analysis**

This analysis was carried out at the level of the site (rather than the collection) because individual collection events were used to calculate the site-level prevalence (*p*) and site-level variance (V_obs_). To calculate the predicted binomial variance, we used the formula V_bin_=[*np*(1-*p*)] where *p* is the mean proportion abnormal at a site, and *n* is the group size, or number of amphibians sampled at each site. We then calculated the natural log of each of these variables and performed a linear regression to estimate the slope (*b*) and intercept (ln(*A*)) of this relationship, which informed us about the degree of aggregation of temporal variation:

ln(V_obs_)=ln(*A*)+*b*ln(V_bin_)

In this relationship, parameter estimates for *A* and *b* equal to 1 suggest a random pattern without aggregation. When both *A* and *b* are >1, the amount of aggregation increases with the mean value (*p*). Estimates of *A>1* and *b*=1 suggest overdispersion without dependence on the mean.

**Supplemental Discussion**

**Minnesota and Vermont Hotspots**

The Minnesota and Vermont sites are not in significant hotspot clusters (Figure 2), because the statistic on which the hotspot cluster determination is calculated from the mean abnormality frequency at a site and those sites near it [[7](#_ENREF_7)] (*Methods and Supplemental Text*). If a site had high mean abnormality frequency and was surrounded by others that also had high frequencies, then it was in a hotspot cluster as defined by the Gi* statistic [[7](#_ENREF_7)] (*Methods and Supplemental Text*). If the mean frequency at a site was lower due to repeated sampling events with low abnormality prevalence, the site was assigned a relatively lower Gi* score, and if it was near sites that also have low abnormality frequencies, the score was lower still. Although the absolute 5% threshold value to determine hotspots in prior studies [[8-10](#_ENREF_8)] is useful for comparison, our cluster-based analysis [[7](#_ENREF_7)] provides a more objective, pattern-based, method for targeting hotspot clusters for study. It remains to be determined how each of these criteria relate to causal mechanisms on the landscape. The lack of a significant hotspot clusters in both Minnesota and Vermont is more likely a result of the mean value at a site being driven down by low abnormality collections within it, and at sites around it, than lack of sampling resolution in these areas.

References

1. USFWS (1999) Standard Operating Procedures for Abnormal Amphibian Monitoring. Available: http://www.fws.gov/contaminants/amphibian/SOPs.html. Accessed 15 April 2013.

2. Legendre P, Legendre L (1998) Developments in Environmental Modeling. Amsterdam: Elsevier, 853 pp.

3. Lichstein J (2006) Multiple regression on distance matrices: a multivariate spatial analysis tool. Plant Ecology 188: 117–131.

4. R Core Development Team (2008) R: A language and environment for statistical computing. Vienna, Austria.

5. Legendre P (1993) Spatial autocorrelation: trouble or new paradigm? Ecology 74: 1659–1673.

6. Hawkins B (2012) Eight (and a half) deadly sins of spatial analysis. Journal of Biogeography 39: 1-9.

7. Getis A, Ord JK (1992) The Analysis of Spatial Association by Use of Distance Statistics. Geographical Analysis 24: 189-206.

8. Lannoo MJ (2008) Malformed Frogs: The Collapse of Aquatic Ecosystems. Berkeley: University of California Press. 270 p.

9. Ouellet M (2000) Amphibian deformities: Current state of knowledge. In: Sparling DW, Linder G, Bishop CA, editors. Ecotoxicology of Amphibians and Reptiles. Pensacola: SETAC Press. pp. 617–646.

10. Johnson PT, Reeves MK, Krest SK, Pinkney AE (2010) A Decade of Deformities: Advances in our Understanding of Amphibian Malformations and their Implications. In: Sparling D, Linder G, Bishop C, Krest S, editors. Ecotoxicology of Amphibians and Reptiles. 2nd ed. Pensacola: SETAC Press. pp. 511-536.
